# Mistranslation can enhance fitness through purging of deleterious mutations

**DOI:** 10.1038/ncomms15410

**Published:** 2017-05-19

**Authors:** Sinisa Bratulic, Macarena Toll-Riera, Andreas Wagner

**Affiliations:** 1 Department of Evolutionary Biology and Environmental Studies, University of Zurich, Winterthurerstrasse 190, Zurich CH-8057, Switzerland; 2 Swiss Institute of Bioinformatics, Quartier Sorge—Bâtiment Génopode, Lausanne 1015, Switzerland; 3 The Santa Fe Institute, Santa Fe, New Mexico 87501, USA

## Abstract

Phenotypic mutations are amino acid changes caused by mistranslation. How phenotypic mutations affect the adaptive evolution of new protein functions is unknown. Here we evolve the antibiotic resistance protein TEM-1 towards resistance on the antibiotic cefotaxime in an *Escherichia coli* strain with a high mistranslation rate. *TEM-1* populations evolved in such strains endow host cells with a general growth advantage, not only on cefotaxime but also on several other antibiotics that ancestral TEM-1 had been unable to deactivate. High-throughput sequencing of *TEM-1* populations shows that this advantage is associated with a lower incidence of weakly deleterious genotypic mutations. Our observations show that mistranslation is not just a source of noise that delays adaptive evolution. It could even facilitate adaptive evolution by exacerbating the effects of deleterious mutations and leading to their more efficient purging. The ubiquity of mistranslation and its effects render mistranslation an important factor in adaptive protein evolution.

## Abstract (web summary)

Mistranslation results in amino acid changes in proteins known as phenotypic mutations and these occur at a much higher rate than DNA mutations. Here, the authors show that mistranslation can increase the response to directional selection by exacerbating the fitness effects of deleterious DNA mutations.

Variation, the essential raw material of all Darwinian evolution, is created by errors in the replication and expression of genetic information. DNA mutations are the best known but not the most frequent source of such errors. More frequent are errors during transcription^1^,^2^, transfer RNA amino-acylation^3^ and translation of messenger RNA into protein^4^. Translation is the most error prone of these processes^4^,^5^,^6^. In *E. coli*, for example, mistranslation occurs at rates of 10^−5^–10^−3^ per codon and thus exceeds the rate of DNA point mutations by a factor of 10^4^ (refs 7, 8). The errors that ribosomes create during mistranslation are sometimes called ‘phenotypic mutations'^8^,^9^. They include missense, read-through or frameshift mutations^5^. Even though they are not as permanent as DNA mutations, phenotypic mutations may influence the evolutionary processes through their sheer abundance. For example, where natural selection stabilizes the function of a protein, mistranslation in combination with natural selection can help reduce a protein's propensity to misfold^5^,^10^,^11^.

Compared with the influence of mistranslation on stabilizing selection, little is known about its influence during directional selection, when proteins acquire new, adaptive functions. On the one hand, mistranslation could reduce the rate of adaptive evolution by natural selection. This is because molecular noise can reduce a population's effective size, which enhances the influence of genetic drift relative to natural selection^12^,^13^. In addition, the evolution of a new protein function may require multiple genetic mutations. This mutational path may lead through mutational intermediates with lower fitness and the fitness loss of such intermediates can be exacerbated by additional, phenotypic mutations that further reduce fitness^14^,^15^,^16^,^17^,^18^. Furthermore, the deleterious effects of mistranslation can alter selection coefficients of sweeping beneficial mutations, thus affecting the efficiency of selective sweeps and the accumulation of variation in large populations^19^,^20^.

On the other hand, mistranslation could also accelerate adaptive evolution, because a beneficial phenotypic mutation can create a high fitness protein from a low fitness genotypic intermediate^9^. For example, under selection for antibiotic resistance, such beneficial phenotypic mutations could enhance the activity of an enzyme that inactivates antibiotics. Under sufficiently strong selection, even few high-fitness proteins may ensure a population's survival and buy the population enough time until genetic mutations make the adaptive change permanent^9^,^21^,^22^. It is unknown whether such ‘stepping stone' proteins exist in the evolution of new protein functions by amino acid-changing mutations, although they may have been involved in the evolution of new protein localization signals via read-through mutations^23^,^24^. In addition, mistranslation could benefit adaptive evolution by exacerbating deleterious effects of mutations. As mistranslation increases the efficiency of purifying selection^11^, it may help eliminate deleterious mutations from standing genetic variation, thus increasing mean population fitness.

To study how protein mistranslation might affect the evolution of a new protein function, we performed laboratory evolution experiments on the antibiotic resistance enzyme TEM-1 *β*-lactamase. Wild-type (‘ancestral') TEM-1 inactivates *β*-lactam antibiotics. It is highly active against penicillins, such as ampicillin. Although it shows negligible activity on cephalosporins, such as cefotaxime, it can evolve high activity against them^25^,^26^,^27^. We experimentally evolved TEM-1 towards activity on cefotaxime in *E. coli* host cells subject to either normal or elevated mistranslation rates and characterized the evolved populations both phenotypically and genotypically via high-throughput sequencing. We show that *TEM-1* populations evolved in error-prone strains have greater genotypic diversity and convey a fitness advantage to their hosts. This advantage is associated with increased purging of deleterious mutations, which can increase mean population fitness.

## Results

### Directed protein evolution of TEM-1

**Figure 1:**
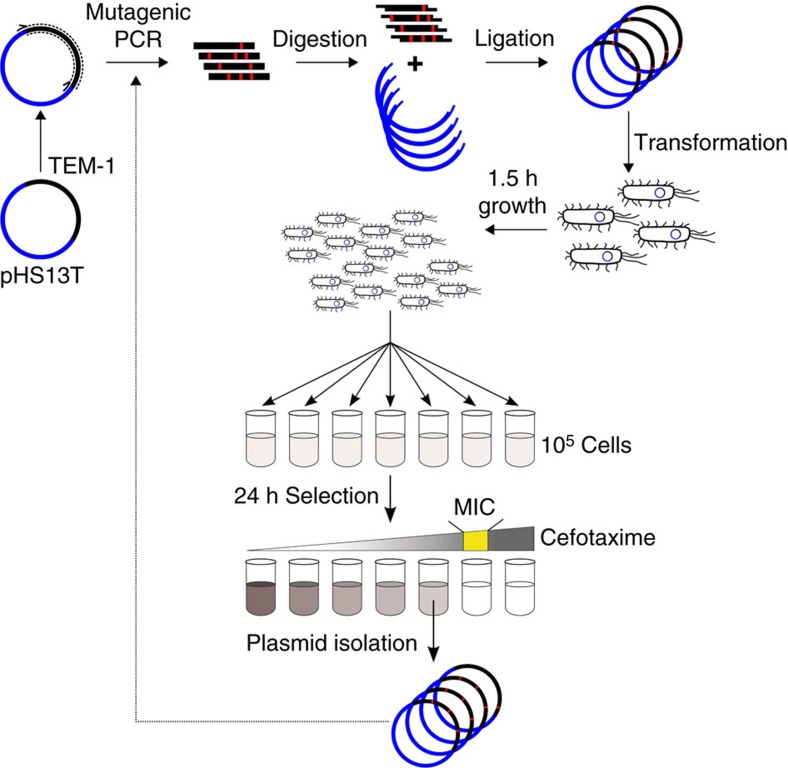
Experimental evolution of TEM-1. In each round (generation) of evolution, we introduced mutations into TEM-1 coding sequences via mutagenic PCR and cloned the resulting mutant sequences into the ancestral plasmid backbone. Single-molecule real-time (SMRT) sequencing of mutated populations revealed that our mutagenesis procedure resulted in ≈0.7 mutations per variant per round. DNA sequencing showed that the mutagenesis procedure was biased towards A→G and T→C substitutions. We transformed populations of plasmids with mutated *TEM-1* into wild-type or error-prone *E. coli* host cells, allowed these cells to grow for 1.5 h and transferred subpopulations of ≈10^5^ cells into liquid LB media with increasing concentrations of cefotaxime, where consecutive media differed by a factor 2 in cefotaxime concentration. After allowing growth and selection to take place for 24 h, we isolated plasmids from the one subpopulation that survived at the highest concentration of cefotaxime and used the collection of *TEM-1* variants isolated from these plasmids as the starting point for the next generation. As a measure of phenotypic evolution, we recorded the MIC of cefotaxime in each round. We evolved four replicate populations per host cell type. After four generations of evolution, we subjected evolved *TEM-1* populations (all time points and all replicates) to SMRT sequencing.

We evolved four independent populations of ≈10^5^ plasmid-borne *TEM-1* variants in two different *E. coli* host strains, a wild-type strain and a strain prone to mistranslation errors, which carries the *rpsD12* mutation^4^,^11^,^28^ in a ribosomal protein-coding gene (Methods). We subjected each population to four cycles (‘generations') of mutation (with a mutation rate of ≈0.7 mutations per variant) and selection^11^,^29^ on increasing concentrations of cefotaxime (Fig. 1). After each generation, we cloned the surviving TEM-1 coding sequence variants into fresh plasmid backbones and transformed them into ancestral (wild-type or error-prone) hosts. Our protocol thus ensures that only the coding sequence of TEM-1 evolves and it allows us to assess the effects of mistranslation errors on protein evolution directly.

**Figure 2:**
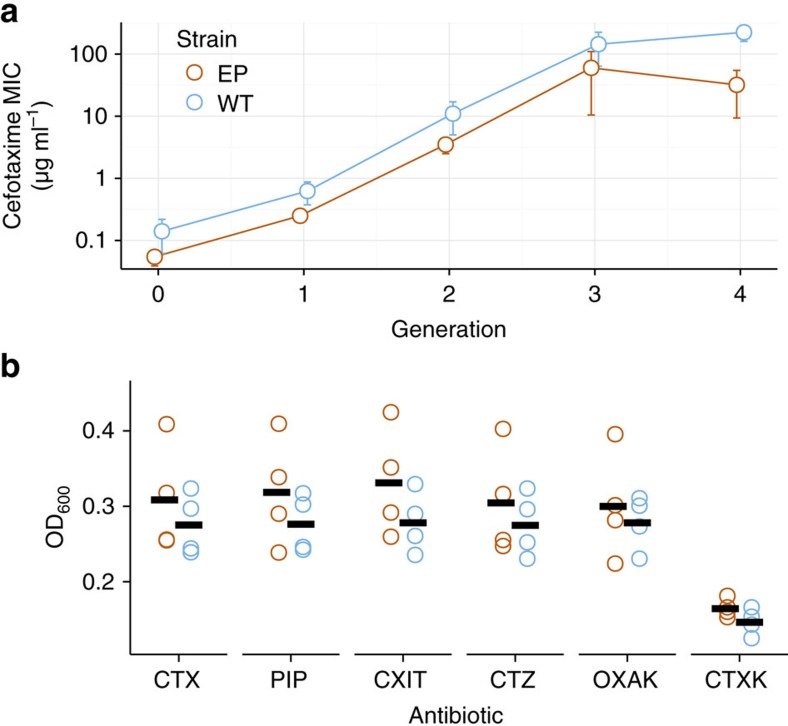
Antibiotic resistance after evolution. (**a**) Mean increase in the MIC of cefotaxime for each of the two hosts during four rounds of evolution on cefotaxime. Circles correspond to means and error bars represent 1 s.d. across four replicate populations. (**b**) Population densities of TEM-1 variants (red=evolved in error-prone hosts; blue=evolved in wild-type hosts) after the fourth round of evolution and after 24 h of growth in wild-type hosts on various *β*-lactam antibiotics (CTX=0.25 μg ml^−1^ cefotaxime, PIP=8 μg ml^−1^ piperacillin, CXIT=0.0625 μg ml^−1^ cefoxitin, CTZ=1 μg ml^−1^ ceftazidime, OXAK=8 μg ml^−1^ oxacillin+0.5 μg ml^−1^ clavulanic acid, CTXK=0.0625 μg ml^−1^ cefotaxime +0.1 μg ml^−1^ clavulanic acid). See Supplementary Fig. 2 for cell densities after expressing evolved TEM-1 populations in both hosts and at all concentrations of all six assayed *β*-lactam antibiotics.

At the start of the evolution experiment (‘generation' 0), the mistranslating host with ancestral TEM-1 showed a lower absolute minimum inhibitory concentration (MIC) for cefotaxime than the wild-type host (Fig. 2a). This is not surprising. Mistranslation can have deleterious effects, because it destabilizes proteins, including TEM-1 (refs 10, 11). In addition, it can have other pleiotropic effects that increase sensitivity to some antibiotics^30^,^31^.

During four rounds of experimental evolution, we observed an up to 2,048-fold increase in the MIC for cefotaxime (Fig. 2a and Supplementary Table 1). If phenotypic mutations can help increase the MIC^9^, one would expect higher MIC in TEM-1 populations that evolved in error-prone hosts. However, this was not the case. The MIC generally stayed lower in error-prone populations (Fig. 2a). In addition, the absolute MIC values followed parallel trajectories in both TEM-1 populations, regardless of whether we assessed their fitness in the wild-type or in an error-prone host (Fig. 2a and Supplementary Figs 1a and 2a). In sum, mistranslation does not facilitate the evolution of high cefotaxime resistance in our experiment.

### Evolution in error-prone hosts leads to a growth advantage

However, an unexpected advantage of evolution under mistranslation revealed itself in a fitness assay that also included other *β*-lactam antibiotics that we had not selected on. Specifically, we transformed *TEM-1* populations from the final round of evolution into wild-type hosts and measured which of these populations grow to higher cell densities within 24 h. First, we found that *TEM-1* populations evolved in error-prone hosts facilitate the host's growth to higher density in the absence of antibiotics (Supplementary Fig. 1b). Second, *TEM-1* populations evolved in error-prone hosts allow the host to grow to higher population densities on low-to-moderate concentrations of cefotaxime (Fig. 2b). Third, they also facilitate growth on the antibiotics piperacillin, cefoxitin, ceftazidime, oxacillin in combination with clavulanic acid and cefotaxime with clavulanic acid (Fig. 2b and Supplementary Fig. 2b–f). Ancestral TEM-1 does not permit any growth on even moderate concentrations of three of these antibiotics (Supplementary Fig. 2a,b,e). The growth advantage persisted when we expressed the *TEM-1* variants in error-prone hosts (Supplementary Fig. 2a–f). This indicates that the advantage is independent of the host in which these variants are expressed and thus specific to the *TEM-1* populations evolved in error-prone hosts. The growth advantage disappears at high concentrations of each antibiotic (Supplementary Fig. 2). Furthermore, the concentration of antibiotics that completely inhibited growth did not differ between populations evolved in wild-type and error-prone hosts (Supplementary Tables 10 and 11). Taken together, these findings suggest that the growth advantage is caused by mutations with modest fitness effects.

To understand the genetic basis of the adaptations we observed, we next used single-molecule real-time sequencing (SMRT^32^) to sequence more than 500 evolved variants per TEM-1 population (see Supplementary Table 2), from each of the 4 generations of evolution.

We first aimed to identify mutations that have sufficiently strong benefits to sweep through a population and attain a frequency exceeding 90% in at least one generation. Such sweeping mutations did indeed occur and they accompanied the exponential increase in resistance to cefotaxime (Supplementary Fig. 3a). Importantly, although mutations sweeping through the population occurred in both hosts, their number was significantly higher for each of the TEM-1 populations evolved in wild-type hosts (four to eight substitutions) than for populations evolved in error-prone hosts (two to three) (two-tailed *t*-test, *t*=3.9337, d.f.=6, *P*=0.0077).

### Mistranslation increases genetic diversity

Previous studies on the evolution of TEM-1 activity on cefotaxime and other cephalosporins revealed five amino acid changes that often occur in combination in laboratory and clinical isolates^14^,^25^,^27^,^33^. The order of appearance and fixation is typically conserved for three of these changes (G238S→E104K→M182T)^14^,^17^,^34^. G238S and E104K jointly improve cefotaxime binding and hydrolysis, but have destabilizing effects that are compensated by M182T^35^. We observed that these three mutations appeared and swept through the population in the canonical order for six of our eight populations (three out of four error prone and three out of four wild type; see Supplementary Fig. 3b). Several other single-nucleotide polymorphisms (SNPs) reached frequencies above 90%, but they did so in only one of the wild-type populations and in none of the error-prone populations (see Supplementary Tables 3 and 4). In sum, although the most strongly selected key mutations are shared between TEM-1 populations evolved in wild-type and error-prone hosts, a greater number of mutations swept to fixation in the wild-type TEM-1 populations.

**Figure 3:**
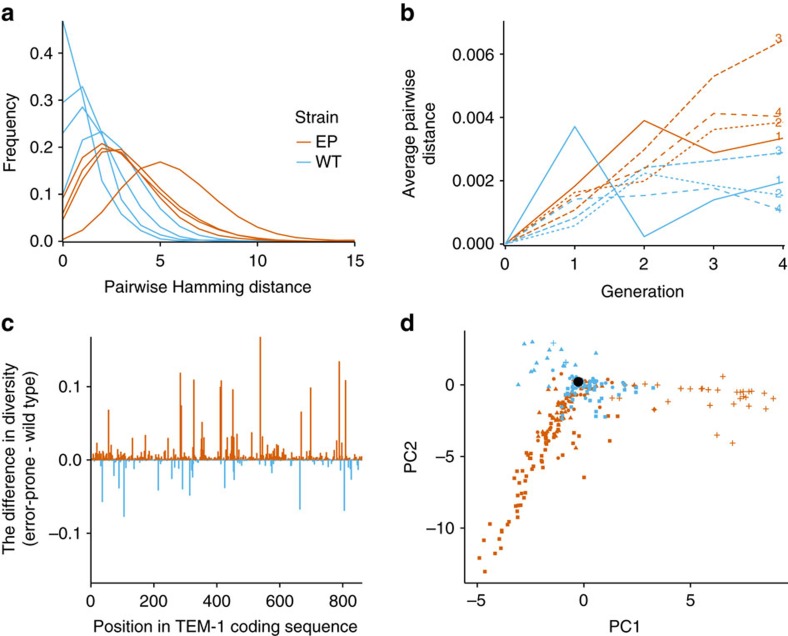
Genetic diversity is greater in *TEM-1* populations evolved in error-prone hosts. (**a**) The distribution of pairwise sequence (Hamming) distances for each of the populations at the end of the experiment (‘generation' 4). Supplementary Fig. 4a shows the distribution of pairwise sequence distances in all four generations. (**b**) Average pairwise sequence distance (see Methods) within evolved populations. Each line corresponds to the diversity trajectory of one of the evolving populations. Each trajectory is shown with a different line pattern and coloured numbers indicate the replicate population. (**c**) The difference in mean nucleotide diversities along the coding sequence for TEM-1 populations evolved in error-prone and wild-type hosts, from the final (fourth) ‘generation' of evolution. We used pairwise alignment positional nucleotide counting (PAPNC) (Methods) to calculate the average diversity at each *TEM-1* nucleotide site for each evolved population. We calculated diversity at a nucleotide site for a given host as the mean of the diversity values from four replicate populations. We then subtracted the mean diversity of the wild-type host from the mean diversity of error-prone hosts, such that values above the horizontal axis (red) correspond to nucleotide sites with higher diversity in error-prone populations. Monomorphic positions are omitted from the plot. (**d**) The distribution of evolved DNA sequences in sequence space. We randomly sampled 200 sequences without replacement from all populations after the fourth generation of evolution, aligned them and then projected the aligned sequences onto two-dimensional space using principal component analysis. The figure shows the first two principal components (PC1 and PC2, explaining 5.3 and 4.6% variability, respectively, see also Supplementary Fig. 6). Each symbol shape corresponds to a sequence, colours correspond to hosts (red=error-prone, blue=wild-type) and different shapes correspond to different replicate populations. The black circle corresponds to the ancestral *TEM-1* sequence.

We next turned our attention to more weakly selected (or possibly neutral) variants, which do not approach fixation during our experiment, and used several complementary approaches to ask which population contained more such variants. First, we calculated all-against-all pairwise sequence (Hamming) distances of evolved *TEM-1* variants for each of the populations. The distribution of these distances is wider and has consistently greater means in *TEM-1* populations evolved in error-prone hosts, and especially so in the third and the fourth round of evolution, suggesting increased diversity under mistranslation (Fig. 3a and Supplementary Fig. 4a).

Second, we computed the genetic diversity in each of the evolving populations. To this end, we first calculated nucleotide diversities using pairwise alignment positional nucleotide counting^36^, which determines the per-population nucleotide diversity as an average of pairwise distances at each position of a multiple DNA sequence alignment. We found that starting in the third and the fourth round of evolution, all four mistranslating populations have a higher mean diversity than any of the wild-type populations (Fig. 3b, two-tailed *t*-test, *t*=3.622, *P*=0.018 for the third round; *t*=3.225, *P*=0.025 for the fourth round). Furthermore, the average higher diversity in mistranslating populations results from many polymorphic sites along the coding sequence of *TEM-1* (Fig. 3c).

Third, we examined the diversity of individual *TEM-1* variants (haplotypes) present in our populations and found that *TEM-1* populations from error-prone hosts harbour more distinct haplotypes than wild-type populations (see Supplementary Fig. 5a,b). In addition, wild-type populations harbour many variants with low frequency and few variants with high frequency (see Supplementary Fig. 5c), whereas mistranslating populations contain more variants at intermediate frequencies.

Finally, to visualize the distribution of haplotypes in sequence space, we sampled 200 sequences from each population and projected these sequence from the high-dimensional sequence space onto two dimensions using principal component analysis (see Methods). This projection showed that *TEM-1* sequences evolved in error-prone hosts are more spread out in sequence space, whereas *TEM-1* sequences evolved in wild-type hosts remain closer to the ancestral *TEM-1* (Fig. 3d and Supplementary Fig. 6). Clustering analysis of sampled haplotypes conveys similar information (Supplementary Fig. 4b). In sum, a greater diversity of weakly selected or neutral mutations exist in *TEM-1* populations evolved in error-prone hosts.

### Mistranslation increases the efficacy of purifying selection

The growth advantage we observed (Fig. 2b and Supplementary Fig. 2) for TEM-1 populations evolved in error-prone hosts could have two causes. Either TEM-1 populations from error-prone hosts harbour fewer deleterious mutations or they harbour more beneficial mutations. To find out which is the case, we performed a one-generation evolution experiment with strong selection on three antibiotics. We started this experiment with populations from the third generation of selection on cefotaxime, because after this time most phenotypic and genotypic evolution had occurred (Figs 2a and 3b, and Supplementary Fig. 3a). We followed our main experimental protocol, except that we selected TEM-1 populations on either piperacillin, cefoxitin or on oxacillin in combination with clavulanic acid. We then SMRT sequenced *TEM-1* variants isolated from a population sample exposed to the highest concentration of antibiotic on which survival was possible (see Supplementary Tables 5–7).

**Figure 4:**
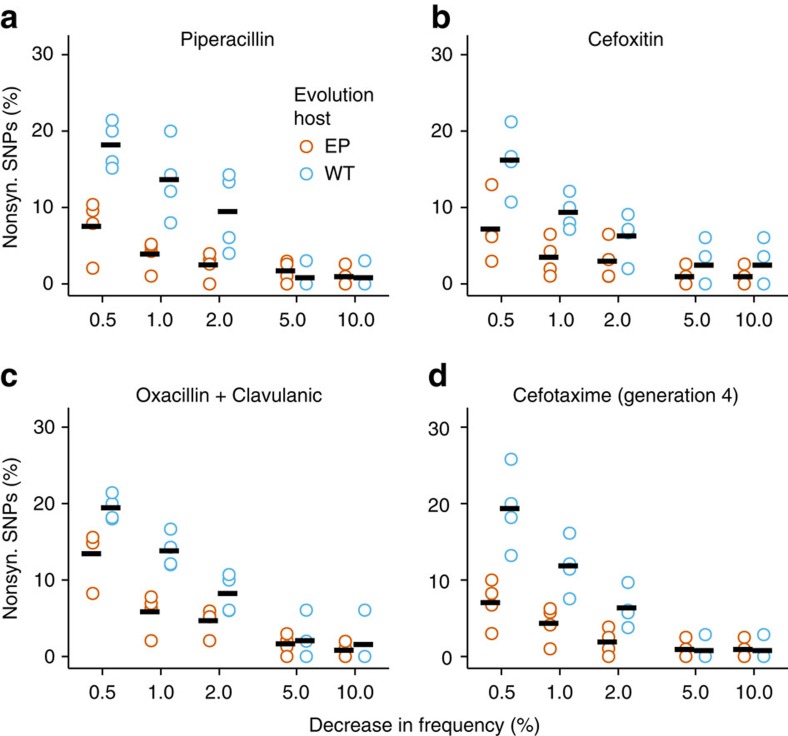
Percentage of deleterious nonsynonymous SNPs in populations after three generations of evolution. The analysis is based on all nonsynonymous SNPs, except the strongly sweeping E104K, M182T and G238S, and shows the percentage of SNPs (vertical axis) that decreases in frequency by at least a given amount (horizontal axis) after a single additional generation of selection on (**a**) piperacillin, (**b**) cefoxitin and (**c**) oxacillin with clavulanic acid in wild-type hosts. (**d**) The percentage of SNPs that decreases in frequency by a given amount after selection of cefotaxime (‘generation' 4 of the evolution experiment). Black lines correspond to the mean across four replicate populations.

We first turned our attention to deleterious mutations. These are mutations that should decrease in frequency under the strong selection regime we imposed. If populations of TEM-1 evolved in error-prone strains harbour fewer deleterious mutations, then a smaller proportion of mutations should decrease in frequency in these populations. This is indeed what we observed on all three antibiotics. For example, the mean fraction of SNPs that decrease in frequency by >1% for selection on piperacillin is 3.9% for TEM-1 populations evolved in error-prone strains, a value that is only one-third as high as the 13.6% of SNPs decreasing frequency for TEM-1 populations evolved in wild-type strains (one-way analysis of variance, *F*_1,6_=13.23, *P*=0.0109; Fig. 4a). This difference disappears as we study more and more strongly deleterious mutations (Fig. 4a, see Fig. 4b for cefoxitin and Fig. 4c for oxacillin with clavulanic acid), showing that it is indeed caused by weakly deleterious mutations. In addition, the same pattern holds for selection on cefotaxime in the fourth generation of our main experiment (Fig. 4d).

We wanted to find out whether SNPs present in error-prone and wild-type populations differ in their effects on protein stability. To this end, we computationally predicted the changes in stability of mutants (ΔΔ*G*_MUT_) after three generations of selection on cefotaxime using FoldX^37^. We found that SNPs present in error-prone populations have a lower ΔΔ*G*_MUT_, that is, they are significantly less destabilizing (two sided Mann–Whitney *U*-test, *U*=14,486, *P*=0.0024). Conversely, SNPs that decrease in frequency upon selection with cefotaxime between generation three and four have a lower ΔΔ*G*_MUT_ in error-prone populations (Supplementary Fig. 9, two-sided Mann–Whitney *U*-test, *U*=9507.5, *P*=0.0046), consistent with the notion that more highly destabilizing SNPs have already been eliminated in error-prone populations before generation 3.

Although some of the SNPs we detected are generally deleterious—they decrease in frequency on all tested antibiotics and in both hosts (Supplementary Fig. 7)—the effects of others are restricted to individual antibiotics. Specifically, up to 5.1% of all deleterious SNPs are specific for oxacillin with clavulanic acid, decreasing in frequency by at least 0.5% on this but no other antibiotics. Thus, despite the promiscuity of TEM-1 lactamase, which can mediate resistance to multiple antibiotics (Supplementary Fig. 2c,d,f), evolution of resistance to specific antibiotics is at least partly mediated by differential purging of deleterious mutations.

A parallel suite of analyses for weakly beneficial mutations (those that increase in frequency by >0.5%) showed that TEM-1 populations from error-prone hosts do not harbour more such mutations (Supplementary Fig. 8). In other words, their growth advantage is mediated by fewer deleterious mutations rather than by more beneficial mutations.

## Discussion

Most random amino acid changes in proteins have effects that are both deleterious and weak^38^,^39^. This fact can help explain the two main observations of our experiments. The first is that strongly beneficial genotypic mutations sweep through both error-prone and wild-type *E. coli* populations, but the number of such sweeps is consistently smaller in error-prone strains (Supplementary Fig. 3a, Supplementary Discussion). In such strains, TEM-1 molecules with strongly beneficial genotypic mutations will more often harbour phenotypic mutations that reduce, on average, the net fitness benefit of the genotypic mutations. In consequence, selective sweeps should become slower or incomplete, just as we observed. Similarly, phenotypic mutations can amplify selection coefficients of deleterious and nearly neutral mutations, thus reducing the amount of hitchhiking^19^. The lower incidence of sweeps, in turn, can explain the greater genotypic diversity of *TEM-1* populations evolved in error-prone strains (Fig. 3). It can also explain the higher abundance of haplotypes with intermediate frequencies (Supplementary Fig. 5). Our observations are consistent with theory predicting that variation can sometimes be maintained in the face of selective sweeps^20^, even though some conditions of our experiment differ from assumptions of the theory (for example, a constant supply of beneficial mutations, no epistasis and an unchanging environment).

The second key observation is that TEM-1 populations evolved in error-prone strains convey a fitness advantage that is associated with a lower incidence of deleterious genotypic variation (Figs 2b and 4, and Supplementary Fig. 2). In an error-prone strain, a TEM-1 molecule with a weakly deleterious genotypic mutation will more often also harbour additional weakly deleterious phenotypic mutations. In combination, the two kinds of mutations will have a more severely deleterious effect than either mutation in isolation. Put differently, in error-prone strains, weakly deleterious genotypic mutations are purged more effectively. This effect is restricted to weakly deleterious mutations, because strongly deleterious genotypic mutations will be eliminated regardless of whether they co-occur with a phenotypic mutation. It can help explain the modest frequency reductions of deleterious alleles we observe (Fig. 4). It can also help explain why the fitness advantages we observe occur at modest but not high antibiotic concentrations (Fig. 2b and Supplementary Fig. 2). Moreover, it is consistent with previous experiments, which showed that mistranslation in combination with stabilizing selection can reduce the amount of genetic polymorphisms in an evolving *TEM-1* population^11^.

In contrast to theoretical postulates^9^ and a previous observation on the evolution of protein localization^24^, we did not observe any evidence that mistranslation creates ‘stepping stone' proteins on a path to highly active TEM-1 (Fig. 2a). Other experimental designs, for example, where adaptive trajectories cross fitness valleys^9^, may be needed to find such proteins. We also note that our experiments used a high mutation rate, such that genotypic mutation alone could, in principle, generate variants with multiple amino acid changes that are adaptive on cefotaxime, which may reduce the benefit of transiently creating beneficial protein variants through mistranslation.

In theory, protein mistranslation can increase the environmental variance of adaptive protein traits and thus reduce their heritability^40^. Moreover, similar to other sources of molecular noise, it can reduce a population's effective size^12^,^13^. Both effects would reduce the efficacy of natural selection. In practice, our experiments showed that mistranslation increases the efficiency of purifying selection. What is more, this effect is detectable even on the short time scales of a laboratory evolution experiment. In the wild, mistranslation could affect protein evolution more profoundly, because its effects can accrue over thousands of generations^41^. In addition, the growth advantage of TEM-1 populations evolved in error-prone *E. coli* strains can be substantial, leading to an up to 15% greater cell density on some antibiotics (Fig. 2b). It would be readily visible to natural selection even in wild populations of modest size. As mistranslation rates vary in nature even within strains of the same species^42^ and can vary more broadly than in our experimental strains^4^,^43^, it is tempting to speculate that prevalent mistranslation rates themselves are a product of adaptive evolution^41^,^42^,^44^,^45^,^46^,^47^,^48^,^49^.

An exciting direction for future work, which could further strenghten our conclusions, is to study strains with mistranslation rates smaller than the wild type (for example, in strain *rpsL141*, refs 4, 50). A caveat to any such analysis is that such strains can experience physiological changes that might affect their evolutionary dynamics for reasons unrelated to the mistranslation rate. Examples include an altered abundance of proteins with non-optimal codons^51^ and medium-dependent changes in growth characteristics^52^.

In sum, we showed that under directional selection for a new antibiotic resistance phenotype, mistranslation can lead to purging of weakly deleterious mutations, which can increase mean population fitness. Under stabilizing selection, mistranslation may decrease mistranslation rates or increase robustness to mistranslation^5^,^11^,^28^. How the effects of mistranslation interact under stabilizing and directional selection remains an important question for future work. As mistranslation may not only have been rampant in early life forms^53^, but is many times more frequent even today than genetic mutations, it remains a potentially important force in the evolution of modern proteins. Our observations raise the possibility that mistranslation may sometimes speed up adaptive evolution by helping to purge deleterious mutations.

## Methods

### Media and antibiotics

We used Difco LB broth (BD) for all experimental steps involving growth and selection. We used SOB media (Sigma) for preparing competent cells and SOC media (SOB media with 20 mM glucose) for recovery of electroporated cells. For antibiotic selection, we used chloramphenicol (Sigma) at 25 and 34 μg ml^−1^, cefotaxime sodium salt (Sigma) at concentrations 0.0078–2,048 μg ml^−1^, cefoxitin sodium salt (Apollo Scientific) at concentrations 0.5–32 μg ml^−1^, oxacillin sodium salt (Sigma) at concentrations 32–2,048 μg ml^−1^, piperacillin (Sigma) at concentrations 64–4,096 μg ml^−1^, potassium clavulanate (Sigma) at concentrations 0.1 μg ml^−1^ (with cefotaxime) and 0.5 μg ml^−1^ (with oxacillin) and ceftazidime hydrate (Sigma) at 0.25–256 μg ml^−1^. We used saline (9 g l^−1^ NaCl) to prepare serial dilutions for library size estimations.

### Strains and plasmids

We used the *E. coli* strain DH5*α* for cloning and the preparation of pre-selection *TEM-1* libraries. The construction of the ribosomal mutant with elevated mistranslation rates, *rpsD12*, and the strain with wild-type mistranslation rates were reported previously^11^. In short, we transfered the *rpsD12* allele into a fresh MG1655 genetic background from the *rpsD12* strain^50^. To this end, we first used recombineering^54^ to integrate a kanamycin resistance cassette flanked by *FRT* sites^54^ into the genome of the *rpsD12* strain downstream of the *rpoA* operon. Next, we used PCR to amplify the region spanning the mutation and the resistance cassette, and integrated the PCR-amplified fragment into an MG1655 background (CGSC#7740). To avoid any nonspecific mutations, we used P1 transduction to transfer the mutation into the MG1655 background. Finally, we removed the *KanR* cassette from P1 transductants using a flipase plasmid pCP20 (ref. 55). We confirmed the final construct by PCR and Sanger sequencing. To construct the strain we call the wild type or normal, we repeated the same cloning procedure using a ‘mock' recombineering construct (a wild-type *rpsD* instead of an *rpsD12* allele). We used pHS13T, which is a high copy number plasmid with a chloramphenicol resistance marker^11^, for evolving TEM-1. To facilitate gel extraction of vector backbones for recloning, we used pHS13K plasmid^11^, which differed from pHS13T by having a *KanR* cassette from pKD4 plasmid^54^ instead of a *TEM-1* gene.

### Electrocompetent cells

To prepare electrocompetent cells, we used glycerol/mannitol density step centrifugation^11^,^56^. In brief, we grew an overnight culture in SOB media. In the morning, we inoculated 2 ml of an overnight culture into a shake flask with 200 ml SOB media and incubated this culture with shaking (250 r.p.m.) at 37 °C, until the OD_600_ reached the values of 0.4–0.6. We stored the culture on ice for 15 min and centrifuged at 1,500 *g* and 4 °C, for 15 min. We resuspended the pellet in 40 ml of cold ddH_2_O and divided the suspension into two 50 ml tubes. We then slowly added 10 ml of ice-cold 20% (w/v) glycerol+1.5% (w/v) mannitol to the bottom of each tube using a 12 ml pipette. We centrifuged the suspension again at 1,500 *g* at 4 °C for 15 min, with acceleration/deceleration set to zero. We aspirated the supernatant and resuspended the pellet in 1 ml of 20% (w/v) glycerol+1.5% (w/v) mannitol. We froze aliquots of competent cells using a dry ice-ethanol bath and stored them in −80 °C.

### Mutagenesis and library cloning

To introduce genetic diversity into TEM-1 populations, we used a mutagenic PCR protocol with nucleoside analogues^57^, as described previously^11^. In short, a 100 μl PCR reaction contained 10 ng of the template plasmid (pHS13T in the first ‘generation' and selected plasmid population in subsequent ‘generations'), with 400 μM dNTPs (Thermo Scientific), 2.5 U *Taq* polymerase (NEB), Thermopol buffer (NEB), 3 μM 8-oxo-GTP, 3 μM dPTP (Trilink Biotechnologies), as well as 400 nM of primers TEM1-F6 and TEM-R6 (Supplementary Table 8). After 25 cycles of PCR, we removed the template plasmid by treating the the PCR product with the restriction enzyme DpnI for 2 h at 37 °C. Subsequently, we inactivated the *Taq* enzyme by adding 0.6 U of proteinase K (Thermo Scientific), incubating for 1 h at 50 °C and then for 15 min at 80 °C. We double digested the mutagenized *TEM-1* pool with 20 U of SacI-HF and HindIII-HF (NEB) for 2 h at 37 °C, followed by an inactivation step for 20 min at 80 °C. We then purified double digested inserts with the QIAprep PCR purification kit (Qiagen). In parallel, we prepared the plasmid backbone by incubating pHS13K with 20 U of SacI-HF and HindIII-HF overnight. We gel purified the double digested vector and used 5 U of Antarctic Phosphatase (NEB) to dephosphorylate it. We prepared ligation reactions by mixing 19 ng of insert (mutagenized *TEM-1* pool), 50 ng of digested and dephosphorylated vector, and 10 U of T4 DNA ligase (NEB). We incubated ligation reactions for 16 h at 4 °C and then for 10 min at 65 °C. We precipitated the ligation product by adding 80 μl of ddH_2_O, 20 μg of glycogen (Thermo Scientific), 50 μl of 7.5 M ammonium acetate (Sigma) and 375 μl of ice-cold absolute ethanol. We incubated the mixture at −20 °C for 20 min, centrifuged for 20 min at 18,000 *g*, washed in 800 μl of 70% cold ethanol, centrifuged and washed again. We dried the pellet, and then resuspended in 15 μl of 2.5 mM Tris-Cl pH 8.5.

### Preselection libraries

We previously established a transformation protocol that allowed us to reproducibly construct preselection libraries with ≈10^5^ clones^11^. Briefly, we mixed 4 μl of the precipitated ligation product products with 80 μl of electrocompetent DH5*α* cells in 0.2 cm electroporation cuvettes (Cell Projects) and electroporated at 15 kV cm^−1^ using a Micropulser electroporator (Bio-Rad). Immediately after electroporation, we added 1 ml of pre-warmed SOC media to transformed cells and transferred the suspension to a 24-well plate. We allowed cells to recover by incubating the plate at 37 °C with shaking at 400 r.p.m. for 1.5 h. After the recovery period, we centrifuged the plate and aspirated the supernatant. We resuspended the cell pellet in 5 ml of LB media supplemented with 34 μg ml^−1^ of chloramphenicol. We used a 50 μl cell suspension aliquot to estimate library size by making serial dilutions in saline and plating on LB agar with 20 μg ml^−1^ chloramphenicol. Through this procedure, we estimated library sizes to lie between 10^5^ and 10^6^ sequences. We expanded the library by incubating transformed cells overnight at 37 °C with shaking at 320 r.p.m. The next morning, we stored 1 ml of the overnight culture as a glycerol stock and used the rest to purify preselection library plasmids with a QIAPrep miniprep kit (Qiagen).

### Selection and control libraries

We transformed 100 μl aliquots of electrocompetent *rpsD12* or wild-type cells with ≈5 ng of purified preselection libraries. The electroporation and recovery conditions were the same as for preselection libraries. We centrifuged the recovered cell suspension for 10 min at 2,800 *g* and resuspended cell pellets in LB media with 34 μg ml^−1^ chloramphenicol. From each of the resuspended libraries, we inoculated ≈10^5^ cells into a twofold dilution series of cefotaxime (the highest concentration of cefotaxime used in the experiment was 2,048 and the lowest 0.0078 μg ml^−1^) in LB media with 34 μg ml^−1^ chloramphenicol. Selection lasted for 24 h with shaking at 320 r.p.m. at 37 °C. We isolated plasmids using the QIAPrep miniprep kit (Qiagen) from the highest concentration of cefotaxime where growth was visible. These plasmids were then used as a starting point for the next generation of evolution.

To estimate mutation rates in each mutagenesis cycle, we constructed one control library for each host strain. These libraries were subject to the same procedure as libraries under selection, except that the selection media contained only 34 μg ml^−1^ chloramphenicol and no cefotaxime. We subjected these control libraries to a single generation of evolution.

### Antibiotic susceptibility assays

We wanted to test the ability of ancestral and evolved TEM-1 populations to confer resistance to different *β*-lactam antibiotics in the two hosts. To this end, we transformed electrocompetent wild-type and mistranslating strains with a pHS13T plasmid carrying ancestral *TEM-1*. We also transformed populations evolved in wild-type hosts into both wild-type and error-prone hosts, and did the same with populations evolved in error-prone hosts. After the recovery period (1.5 h in SOC media, at 37 °C, shaking at 400 r.p.m.), we centrifuged cultures for 10 min at 2,200 *g*, aspirated the supernatant and resuspended the cell pellet in 4.5 ml of LB supplemented with 34 μg ml^−1^ chloramphenicol. We grew these cultures for 5 h at 37 °C with shaking at 320 r.p.m. and then stored them as glycerol stocks at −80 °C.

On the morning of the susceptibility assay, we scraped frozen cultures and inoculated them in 96 deep-well plates (Nunc) with 1.1 ml of LB and 34 μg ml^−1^ chloramphenicol. We grew these cultures for ≈5 h, measured their OD_600_ and diluted them to an OD_600_ of 0.01. We inoculated 10 μl of the diluted cultures into 96-well plates with 190 μl of LB supplemented with chloramphenicol and a series of twofold dilutions of cefotaxime, ceftazidime, cefoxitin, piperacillin, cefotaxime with clavulanic acid (0.1 μg ml^−1^) and oxacillin with clavulanic acid (0.5 μg ml^−1^). Concentrations of antibiotics we used in the experiment are given in the Supplementary Table 9. We incubated these plates at 37 °C without shaking and measured OD_600_ after 24 h.

### SMRT sequencing

We prepared and barcoded experimental populations for SMRT sequencing using a two-step PCR procedure^11^. First, we used a 25-cycle PCR with the Phusion polymerase, to amplify the coding region of *TEM-1* with TEM1FS-F and TEM1FS-R primers (Supplementary Table 8). We gel purified PCR products and used them as templates for a second PCR with barcoded primers BCXX and ELP (see Supplementary Table 8 for primer and barcode sequences), based on 6 bp-long barcodes described in ref. 58. We purified PCR products with a QIAprep PCR purification kit (Qiagen). We then used the Agilent 2200 TapeStation System (Agilent Technologies) to check the quality and concentrations of amplicons in each barcoded library. To account for sequencing and library preparation errors, we amplified and barcoded an additional library from an ancestral *TEM-1* sequence. Finally, we combined 20 ng of DNA from each library, to create two amplicon pools for sequencing. The first pool contained amplicons from the first two generations and the second pool contained amplicons from the last two generations of evolution.

We produced a SMRTbell library from the amplicon pool with the DNA Template Prep Kit 2.0 (250 bp–3 Kb) (Pacific Biosciences). We created the SMRTbell template by ligating blunt end adapters to amplicons. We used a DNA/Polymerase P4 binding kit (Pacific Biosciences) according to the manufacturer's instructions, to create a ready-to-sequence SMRTbell–polymerase complex. We programmed the Pacific Biosciences RS2 instrument to sequence each amplicon pool on one SMRT cell v3.0 (Pacific Biosciences), using P4/C2 chemistry, the magnetic bead loading method and taking two movies of 180 min for each cell.

### Primary data analysis

We used a previously described pipeline^11^ for primary analysis of the SMRT sequence data. In short, we assembled consensus reads of evolved *TEM-1* variants (reads of insert) from raw subreads using the SMRTAnalysis v2.3 package^59^. We filtered reads of insert according to (a) the minimum number of full pass subreads (4), (b) the minimum predicted consensus accuracy (0.9) and (c) insert length (850–1,200 bp). With a mean number of ≈12.3 passes per read of insert, this procedure resulted in 51,133 reads, with a mean read length of 980 bp and an average read quality of ≈0.9925.

We mapped reads to the reference *TEM-1* sequence (GenBank accession code: KT391064) using BLASR^60^ with a minimum accuracy of 0.9 and a minimum mapped length of 850 bp. The resulting total number of mapped reads was 51,034, with the average mapped read length being 975 bp. The mean mapped subread concordance^61^ was 0.976. We further filtered mapped reads to include only those reads with average Phred quality >20 and spanning the entire coding region of the *TEM-1* reference in the alignment. We excluded a small fraction (typically 1–2% per library) of sequences which lacked a stop codon or had an internal stop codon. We demultiplexed the filtered set of reads according to their barcodes, using custom Python scripts based on the pbcore module (http://github.com/PacificBiosciences/pbcore). The final set of *TEM-1* variants (27,782) contained only sequences whose barcodes perfectly matched those we used during library preparation. Computer code is available on request.

Similar to our previous study^11^, we ignored indels and focused our analysis on point mutations. We considered a mismatch of a *TEM-1* sequence read to the reference *TEM-1* sequence a true SNP only if its Phred quality score was above 20 (see Supplementary Table 2 for summary statistics).

### Predicting the stability effects of mutations

We computationally predicted changes of stability caused by SNPs present in evolved populations using FoldX^37^ (ver 4.0, http://foldxsuite.crg.eu). First, we introduced the three canonical SNPs that facilitate hydrolysis of cefotaxime (E104K, M182T and G238S) into the structure of TEM-1 (PDBid: 1XPB) using the BuildModel function of FoldX. Second, using the FoldX PositionScan function, we introduced SNPs present in evolved populations into the structure one by one. Finally, we calculated the difference in stability of a mutant relative to the starting structure as ΔΔ*G*_MUT_.

### Genetic diversity calculations

We used the pairwise alignment positional nucleotide counting approach^36^ to calculate the per-site and average genetic diversity in populations of *TEM-1*. Specifically, we first calculated the genetic diversity at site *j* as:


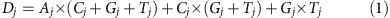


where *A*_*j*_, *C*_*j*_, *G*_*j*_ and *T*_*j*_ are the numbers of bases A, C, G and T, respectively, at site *j* in the alignment. The per-site distance is expressed as:


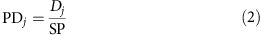


where 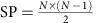 and *N* is the number of nucleotides at position *j*. The average per-site distance is:


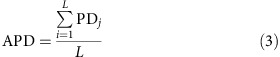


### Principal component analysis

We randomly sampled 200 sequences without replacement from populations in the fourth generation of evolution. We assembled these sequences into a multiple sequence alignment and removed all the columns that correspond to positions at which SNPs had swept through the population (that is, achieved a frequency exceeding 90%) in at least one population. We used the dudi.pca function from the ade4 1.7–4 package^62^ to decompose the alignment and visualized the projection onto the first three principal components using ggplot2 2.1.0 (ref. 63) in *R* 3.2.5 (ref. 64).

### Data availability

Sequence data from the study have been deposited in the GenBank Nucleotide database with the accession codes KY713624 to KY727554 and KY727555 to KY741536.

## Additional information

**How to cite this article:** Bratulic, S. *et al*. Mistranslation can enhance fitness through purging of deleterious mutations. *Nat. Commun.* **8,** 15410 doi: 10.1038/ncomms15410 (2017).

**Publisher's note:** Springer Nature remains neutral with regard to jurisdictional claims in published maps and institutional affiliations.

## Supplementary Material

Supplementary Information Supplementary Figures, Supplementary Tables, Supplementary Discussion and Supplementary References

## References

[b1] Gout J.-F., Thomas W. K., Smith Z., Okamoto K. & Lynch M. Large-scale detection of *in vivo* transcription errors. Proc. Natl Acad. Sci. USA 110, 18584–18589 (2013). 24167253 10.1073/pnas.1309843110 PMC3832031

[b2] Traverse C. C. & Ochman H. Conserved rates and patterns of transcription errors across bacterial growth states and lifestyles. Proc. Natl Acad. Sci. USA 113, 3311–3316 (2016). 26884158 10.1073/pnas.1525329113 PMC4812759

[b3] Swanson R. . Accuracy of *in vivo* aminoacylation requires proper balance of tRNA and aminoacyl-tRNA synthetase. Science 242, 1548–1551 (1988). 3144042 10.1126/science.3144042

[b4] Kramer E. B. & Farabaugh P. J. The frequency of translational misreading errors in *E. coli* is largely determined by tRNA competition. RNA 13, 87–96 (2007). 17095544 10.1261/rna.294907 PMC1705757

[b5] Drummond D. A. & Wilke C. O. The evolutionary consequences of erroneous protein synthesis. Nat. Rev. Genet. 10, 715–724 (2009). 19763154 10.1038/nrg2662 PMC2764353

[b6] Meyerovich M., Mamou G. & Ben-Yehuda S. Visualizing high error levels during gene expression in living bacterial cells. Proc. Natl Acad. Sci. USA 107, 11543–11548 (2010). 20534550 10.1073/pnas.0912989107 PMC2895060

[b7] Drake J., Charlesworth B., Charlesworth D. & Crow J. F. Rates of spontaneous mutation. Genetics 148, 1667–1686 (1998). 9560386 10.1093/genetics/148.4.1667 PMC1460098

[b8] Bürger R., Willensdorfer M. & Nowak M. A. Why are phenotypic mutation rates much higher than genotypic mutation rates? Genetics 172, 197–206 (2006). 16143614 10.1534/genetics.105.046599 PMC1456147

[b9] Whitehead D. J., Wilke C. O., Vernazobres D. & Bornberg-Bauer E. The look-ahead effect of phenotypic mutations. Biol. Direct 3, 18 (2008). 18479505 10.1186/1745-6150-3-18 PMC2423361

[b10] Drummond D. A. & Wilke C. O. Mistranslation-induced protein misfolding as a dominant constraint on coding-sequence evolution. Cell 134, 341–352 (2008). 18662548 10.1016/j.cell.2008.05.042 PMC2696314

[b11] Bratulic S., Gerber F. & Wagner A. Mistranslation drives the evolution of robustness in TEM-1 *β*-lactamase. Proc. Natl Acad. Sci. USA 112, 12758–12763 (2015). 26392536 10.1073/pnas.1510071112 PMC4611672

[b12] Wang Z. & Zhang J. Impact of gene expression noise on organismal fitness and the efficacy of natural selection. Proc. Natl Acad. Sci. USA 108, E67–E76 (2011). 21464323 10.1073/pnas.1100059108 PMC3080991

[b13] Mineta K., Matsumoto T., Osada N. & Araki H. Population genetics of non-genetic traits: Evolutionary roles of stochasticity in gene expression. Gene 562, 16–21 (2015). 25752289 10.1016/j.gene.2015.03.011

[b14] Weinreich D. M., Delaney N. F., Depristo M. A. & Hartl D. L. Darwinian evolution can follow only very few mutational paths to fitter proteins. Science 312, 111–114 (2006). 16601193 10.1126/science.1123539

[b15] Tokuriki N., Stricher F., Serrano L. & Tawfik D. S. How protein stability and new functions trade off. PLoS Comput. Biol. 4, e1000002 (2008). 18463696 10.1371/journal.pcbi.1000002 PMC2265470

[b16] Kvitek D. J. & Sherlock G. Reciprocal sign epistasis between frequently experimentally evolved adaptive mutations causes a rugged fitness landscape. PLoS Genet. 7, e1002056 (2011). 21552329 10.1371/journal.pgen.1002056 PMC3084205

[b17] Salverda M. L. M. . Initial mutations direct alternative pathways of protein evolution. PLoS Genet. 7, e1001321 (2011). 21408208 10.1371/journal.pgen.1001321 PMC3048372

[b18] Gong L. I., Suchard M. A. & Bloom J. D. Stability-mediated epistasis constrains the evolution of an influenza protein. eLife 2013, e00631 (2013). 10.7554/eLife.00631 PMC3654441 23682315

[b19] Good B. H. & Desai M. M. Deleterious passengers in adapting populations. Genetics 198, 1183–1208 (2014). 25194161 10.1534/genetics.114.170233 PMC4224160

[b20] Desai M. M. & Fisher D. S. Beneficial mutation-selection balance and the effect of linkage on positive selection. Genetics 176, 1759–1798 (2007). 17483432 10.1534/genetics.106.067678 PMC1931526

[b21] Modeling B., Masel J. & Bergman A. The evolution of the evolvability properties of the yeast prion [PSI+]. Evolution 57, 1498–1512 (2003). 12940355 10.1111/j.0014-3820.2003.tb00358.x

[b22] Griswold C. K. & Masel J. Complex adaptations can drive the evolution of the capacitor [PSI+], even with realistic rates of yeast sex. PLoS Genet. 5, e1000517 (2009). 19521499 10.1371/journal.pgen.1000517 PMC2686163

[b23] Giacomelli M. G., Hancock A. S. & Masel J. The conversion of 3′ UTRs into coding regions. Mol. Biol. Evol. 24, 457–464 (2007). 17099057 10.1093/molbev/msl172 PMC1808353

[b24] Yanagida H. . The evolutionary potential of phenotypic mutations. PLOS Genet. 11, e1005445 (2015). 26244544 10.1371/journal.pgen.1005445 PMC4526572

[b25] Palzkill T. & Botstein D. Identification of amino acid substitutions that alter the substrate specificity of TEM-1 *β*-lactamase. J. Bacteriol. 174, 5237–5243 (1992). 1644749 10.1128/jb.174.16.5237-5243.1992 PMC206357

[b26] Zaccolo M. & Gherardi E. The effect of high-frequency random mutagenesis on in vitro protein evolution: a study on TEM-1 *β*-lactamase. J. Mol. Biol. 285, 775–783 (1999). 9878443 10.1006/jmbi.1998.2262

[b27] Orencia M. C., Yoon J. S., Ness J. E., Stemmer W. P. C. & Stevens R. C. Predicting the emergence of antibiotic resistance by directed evolution and structural analysis. Nat. Struct. Biol. 8, 238–242 (2001). 11224569 10.1038/84981

[b28] Manickam N., Nag N., Abbasi A., Patel K. & Farabaugh P. J. Studies of translational misreading *in vivo* show that the ribosome very efficiently discriminates against most potential errors. RNA 20, 9–15 (2014). 24249223 10.1261/rna.039792.113 PMC3866648

[b29] Barlow M. & Hall B. G. Predicting evolutionary potential: *in vitro* evolution accurately reproduces natural evolution of the TEM *β*-lactamase. Genetics 160, 823–832 (2002). 11901104 10.1093/genetics/160.3.823 PMC1462021

[b30] Kohanski M. A., Dwyer D. J., Wierzbowski J., Cottarel G. & Collins J. J. Mistranslation of membrane proteins and two-component system activation trigger antibiotic-mediated cell death. Cell 135, 679–690 (2008). 19013277 10.1016/j.cell.2008.09.038 PMC2684502

[b31] Bacher J. M., de Crécy-Lagard V. & Schimmel P. R. Inhibited cell growth and protein functional changes from an editing-defective tRNA synthetase. Proc. Natl Acad. Sci. USA 102, 1697–1701 (2005). 15647356 10.1073/pnas.0409064102 PMC547871

[b32] Eid J. . Real-time DNA sequencing from single polymerase molecules. Science 323, 133–138 (2009). 19023044 10.1126/science.1162986

[b33] Salverda M. L. M., de Visser J. A. G. & Barlow M. Natural evolution of TEM-1 *β*-lactamase: experimental reconstruction and clinical relevance. FEMS Microbiol. Rev. 34, 1015–1036 (2010). 20412308 10.1111/j.1574-6976.2010.00222.x

[b34] Schenk M. F. . Role of pleiotropy during adaptation of TEM-1 *β* -lactamase to two novel antibiotics. Evol. Appl. 8, 248–260 (2015). 25861383 10.1111/eva.12200 PMC4380919

[b35] Sideraki V., Huang W., Palzkill T. & Gilbert H. F. A secondary drug resistance mutation of TEM-1 *β*-lactamase that suppresses misfolding and aggregation. Proc. Natl Acad. Sci. USA 98, 283–288 (2001). 11114163 10.1073/pnas.011454198 PMC14582

[b36] Shao W. . PAPNC, a novel method to calculate nucleotide diversity from large scale next generation sequencing data. J. Virol. Methods 203, 73–80 (2014). 24681054 10.1016/j.jviromet.2014.03.008 PMC4104926

[b37] Schymkowitz J. . The FoldX web server: an online force field. Nucleic Acids Res. 33, W382–W388 (2005). 15980494 10.1093/nar/gki387 PMC1160148

[b38] Tokuriki N., Stricher F., Schymkowitz J., Serrano L. & Tawfik D. S. The stability effects of protein mutations appear to be universally distributed. J. Mol. Biol. 369, 1318–1332 (2007). 17482644 10.1016/j.jmb.2007.03.069

[b39] Firnberg E., Labonte J. W., Gray J. J. & Ostermeier M. A comprehensive, high-resolution map of a gene's fitness landscape. Mol. Biol. Evol. 31, 1581–1592 (2014). 24567513 10.1093/molbev/msu081 PMC4032126

[b40] Lynch M. & Walsh B. Genetics and Analysis of Quantitative Traits Sinauer (1998).

[b41] Rajon E. & Masel J. Evolution of molecular error rates and the consequences for evolvability. Proc. Natl Acad. Sci. USA 108, 1082–1087 (2011). 21199946 10.1073/pnas.1012918108 PMC3024668

[b42] Mikkola R. & Kurland C. G. Selection of laboratory wild-type phenotype from natural isolates of *Escherichia coli* in chemostats. Mol. Biol. Evol. 9, 394–402 (1992). 1584010 10.1093/oxfordjournals.molbev.a040731

[b43] Mikkola R. & Kurland C. Is there a unique ribosome phenotype for naturally occurring *Escherichia coli*? Biochimie 73, 1061–1066 (1991). 1720663 10.1016/0300-9084(91)90148-t

[b44] Jones T. E., Alexander R. W. & Pan T. Misacylation of specific nonmethionyl tRNAs by a bacterial methionyl-tRNA synthetase. Proc. Natl Acad. Sci. USA 108, 6933–6938 (2011). 21482813 10.1073/pnas.1019033108 PMC3084089

[b45] Miranda I. . *Candida albicans* CUG mistranslation is a mechanism to create cell surface variation. mBio 4, e00285–13 (2013). 23800396 10.1128/mBio.00285-13 PMC3697807

[b46] Li L. . Naturally occurring aminoacyl-tRNA synthetases editing-domain mutations that cause mistranslation in *Mycoplasma* parasites. Proc. Natl Acad. Sci. USA 108, 9378–9383 (2011). 21606343 10.1073/pnas.1016460108 PMC3111296

[b47] Li L. . Leucyl-tRNA synthetase editing domain functions as a molecular rheostat to control codon ambiguity in *Mycoplasma* pathogens. Proc. Natl Acad. Sci. USA 110, 3817–3822 (2013). 23431144 10.1073/pnas.1218374110 PMC3593832

[b48] Javid B. . Mycobacterial mistranslation is necessary and sufficient for rifampicin phenotypic resistance. Proc. Natl Acad. Sci. USA 111, 1132–1137 (2014). 24395793 10.1073/pnas.1317580111 PMC3903211

[b49] Su H.-W. . The essential mycobacterial amidotransferase GatCAB is a modulator of specific translational fidelity. Nat. Microbiol. 1, 16147 (2016). 27564922 10.1038/nmicrobiol.2016.147

[b50] Ballesteros M., Fredriksson A., Henriksson J. & Nyström T. Bacterial senescence: protein oxidation in non-proliferating cells is dictated by the accuracy of the ribosomes. EMBO J. 20, 5280–5289 (2001). 11566891 10.1093/emboj/20.18.5280 PMC125621

[b51] Pelchovich G. . Ribosomal mutations affecting the translation of genes that use non-optimal codons. FEBS J. 281, 3701–3718 (2014). 24966114 10.1111/febs.12892

[b52] Paulander W., Maisnier-Patin S. & Andersson D. I. The fitness cost of streptomycin resistance depends on *rpsL* mutation, carbon source and RpoS (*σ*^*S*^). Genetics 183, 539–546 1SI-2SI (2009). 19652179 10.1534/genetics.109.106104 PMC2766315

[b53] Woese C. R. On the evolution of the genetic code. Proc. Natl Acad. Sci. USA 54, 1546–1552 (1965). 5218910 10.1073/pnas.54.6.1546 PMC300511

[b54] Datsenko K. A. & Wanner B. L. One-step inactivation of chromosomal genes in *Escherichia coli* K-12 using PCR products. Proc. Natl Acad. Sci. USA 97, 6640–6645 (2000). 10829079 10.1073/pnas.120163297 PMC18686

[b55] Cherepanov P. P. & Wackernagel W. Gene disruption in *Escherichia coli*: TcR and KmR cassettes with the option of Flp-catalyzed excision of the antibiotic-resistance determinant. Gene 158, 9–14 (1995). 7789817 10.1016/0378-1119(95)00193-a

[b56] Warren D. J. Preparation of highly efficient electrocompetent *Escherichia coli* using glycerol/mannitol density step centrifugation. Anal. Biochem. 413, 206–207 (2011). 21362398 10.1016/j.ab.2011.02.036

[b57] Zaccolo M., Williams D. M., Brown D. M. & Gherardi E. An approach to random mutagenesis of DNA using mixtures of triphosphate derivatives of nucleoside analogues. J. Mol. Biol. 255, 589–603 (1996). 8568899 10.1006/jmbi.1996.0049

[b58] Chubiz L. M., Lee M.-C., Delaney N. F. & Marx C. J. FREQ-Seq: a rapid, cost-effective, sequencing- based method to determine allele frequencies directly from mixed populations. PLoS ONE 7, e47959 (2012). 23118913 10.1371/journal.pone.0047959 PMC3485326

[b59] Pacific Biosciences DevNet. Available at http://www.smrtcommunity.com/DevNet (2015).

[b60] Chaisson M. J. & Tesler G. Mapping single molecule sequencing reads using basic local alignment with successive refinement (BLASR): application and theory. BMC Bioinformatics 13, 238 (2012). 22988817 10.1186/1471-2105-13-238 PMC3572422

[b61] Kim K. E. . Long-read, whole-genome shotgun sequence data for five model organisms. Sci. Data 1, 140045 (2014). 25977796 10.1038/sdata.2014.45 PMC4365909

[b62] Dray S. & Dufour A. B. The ade4 package: implementing the duality diagram for ecologists. J. Stat. Softw. 22, 1–20 (2007).

[b63] Wickham H. ggplot2: Elegant Graphics for Data Analysis Springer New York (2009).

[b64] R Development Core Team. R: A language and environment for statistical computing http://www.r-project.org (2008).

