# Supplementary material for: Mistranslation can enhance fitness through purging of deleterious mutations
Source: Nat Commun. 2017 May 19;8:15410. doi: 10.1038/ncomms15410 (PMC5454534; doi:10.1038/ncomms15410)
Supplement: Supplementary Information — Supplementary Figures, Supplementary Tables, Supplementary Discussion and Supplementary References [file ncomms15410-s1.pdf]

Supplementary Discussion

Selective sweeps

In a previous study, we had observed that most SNPs reaching intermediate and high frequencies appeared in the signal peptide of TEM-1.[1] This suggested an important role for the evolution of gene expression in adaptation to elevated mistranslation rates. In contrast, in our present experiments we found that most SNPs reaching intermediate and high frequencies occured in the structural part of TEM-1 (Supplementary Tables 3 and 4). The only two fixed SNPs in the signaling peptide were I13T, and the synonymous change CTT21CTG. Both of these substitutions were fixed only in wild-type populations. Scarcity of changes in this region reflects weak selection for increased or decreased expression of TEM-1.

When studying SNPs appearing in the structural part of TEM-1, we only observed three of the four substitutions that often occur in combination in laboratory and clinical isolates.[2–4] Of the four substitutions (A42G, E104K, M182T, and G238S), we did not observe A42G. A42G may stabilize the active site of TEM-1,[3] and its absence can be compensated for by other stabilizing substitutions.[5] Indeed, we found T265M and other stabilizing substitutions present at high frequencies in evolved populations (Supplementary Table 3).

In our experiments, the exponential increase in cefotaxime MIC was accompanied by selective sweeps of G238G, E104K and M182T in six out of eight experimental populations (Supplementary Fig. 3a,b), regardless of the rate of mistranslation. M182T was absent from only one of the wild-type populations (Supplementary Fig. 3b, WT 3), but its stabilizing effect may have been compensated by H153R[6] and A224V[7] which occurred in this population (Supplementary Table 3). In addition, in one error-prone population M182T appeared but never reached a frequency above 90% (Supplementary Fig. 3C, EP 3). This population harbored H153R, as well as S286G, which can also stabilize TEM-1.[8]

If mistranslation increased the strength of selection for protein stability[1] in our experiments, we would expect stabilizing SNPs to reach higher frequencies in mistranslating compared to wild-type populations. However, out of all stabilizing SNPs that became fixed in wild-type populations (I47V, N100D, I208M, T265M, and K288E), I47V is the only one found at high frequency in a mistranslating population, and it occurs in only one such population ($\approx 88\%$) (Supplementary Table 3). The finding that stabilizing SNPs are not present at high frequencies in mistranslating populations suggests that selection for resistance to cefotaxime is much stronger than selection for stability in mistranslating hosts. For example, G238S is a crucial early step in the evolution of resistance to cefotaxime.[4] However, G238S destabilizes TEM-1 and this slows down the accumulation of other nonsynonymous SNPs in mistranslating conditions. Even if potentially stabilizing SNPs occur in the population, their fixation is prevented by strong selection that drives alleles with G238S (in the first round of evolution) and E104K (in the second) to high frequency.

The finding that stabilizing SNPs found in wild-type populations lack parallelism, i.e. that these SNPs fix only in one replicate population, supports the claim that these SNPs have weak beneficial effects at best under selection for resistance against cefotaxime.

That strong selection for resistance against cefotaxime rather than for increased stability or expression drives most adaptive changes, is supported by an additional finding: No synonymous SNP reaches high frequency in TEM-1 populations evolved in mistranslating hosts, even though some such SNPs could reduce destabilizing effects of mistranslation by increasing translational accuracy. Taken together, these results suggested that selective sweeps occuring in our experiment are dominated by selection for high activity against cefotaxime.

Supplementary Figures

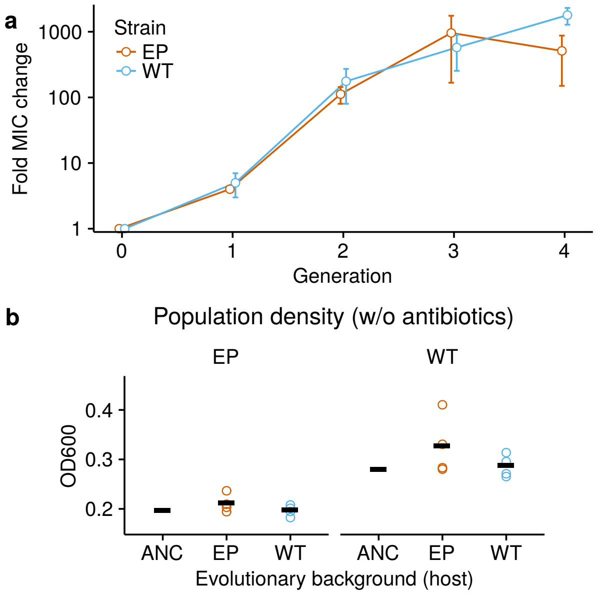

Supplementary Figure 1: Phenotypic evolution of TEM-1. **a**, Relative increase in cefotaxime resistance during the evolution experiment. To get the relative increase in MIC on cefotaxime, we divided the MIC of a population with the MIC of the ancestral TEM-1 allele carried by the same type of host (error-prone or wild-type). Points correspond to means of four replicate populations, and error bars refer to standard deviations. **b**, Optical density of evolved populations in media without antibiotics. We transformed each evolved population into error-prone (EP) and wild-type (WT) hosts, and determined its optical density after 24 h of growth in media with no antibiotics. Circles correspond to the mean optical density of replicate populations (each mean is based on 38 measurements). Each black horizontal bar corresponds to mean optical density across four replicate populations. horizontal axis labels: ANC:ancestral TEM-1, EP: TEM-1 populations evolved in error-prone hosts; WT: TEM-1 populations evolved in error-prone hosts.

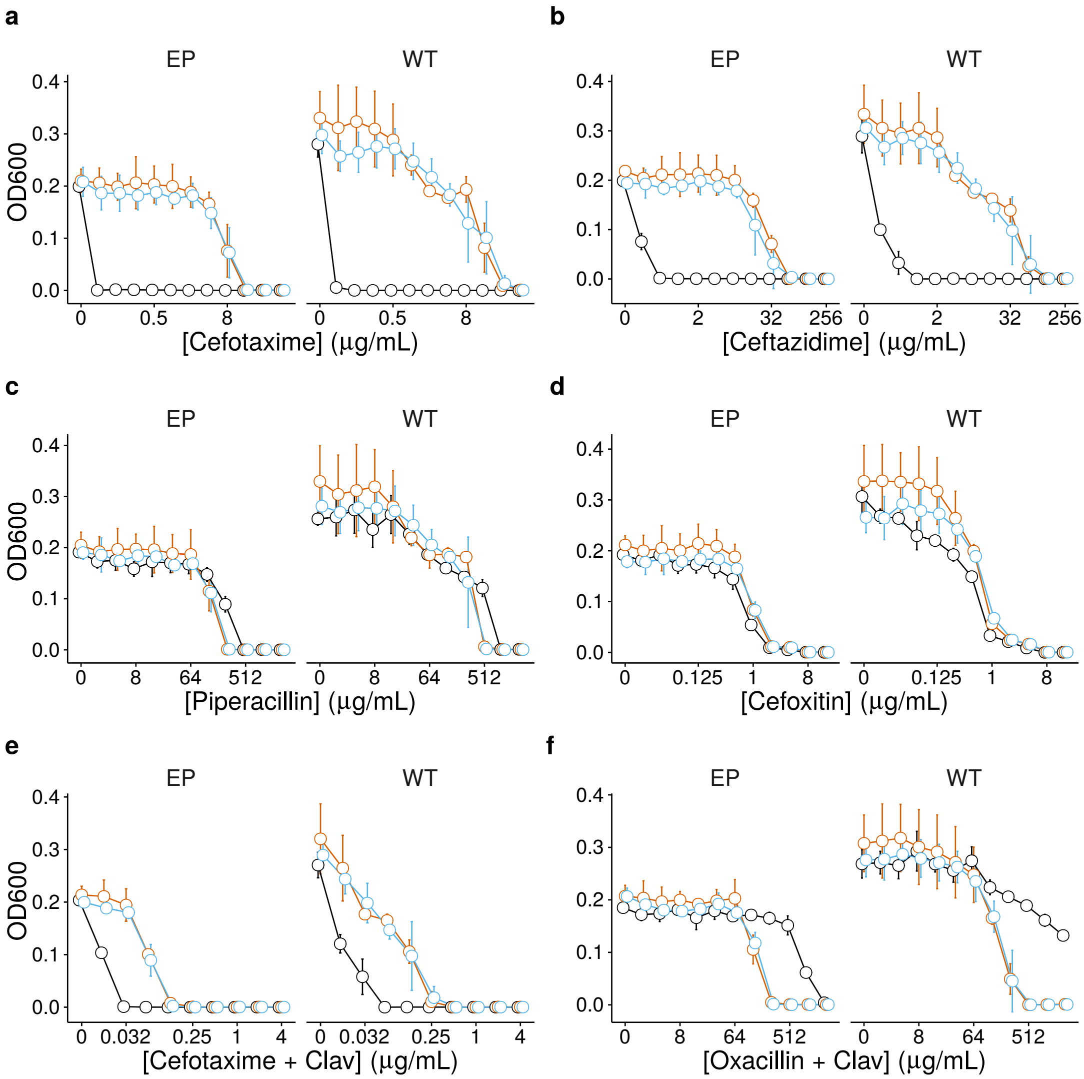

Supplementary Figure 2: Optical densities (OD_{600}) of evolved populations measured in media with different β-lactam antibiotics and β-lactamase inhibitors (Clav = clavulanic acid). Each population that had evolved in wild-type hosts (blue) and error-prone hosts (red) was expressed in both wild-type (WT) and error-prone (EP) hosts. We used ancestral TEM-1 as a control in these experiments (black). Transformed cells were allowed to recover and then exposed to LB media with different concentrations of β-lactam antibiotics. Optical density was measured at 600 nm after $\approx$ 24 hours. Optical density was computed as a mean from at least four independent experiments, and circles correspond to means across four populations. Error bars refer to standard deviations across four populations. Clavulanic acid concentration was 0.1 μg/mL in combination with cefotaxime and 0.5 μg/mL in combination with oxacillin.

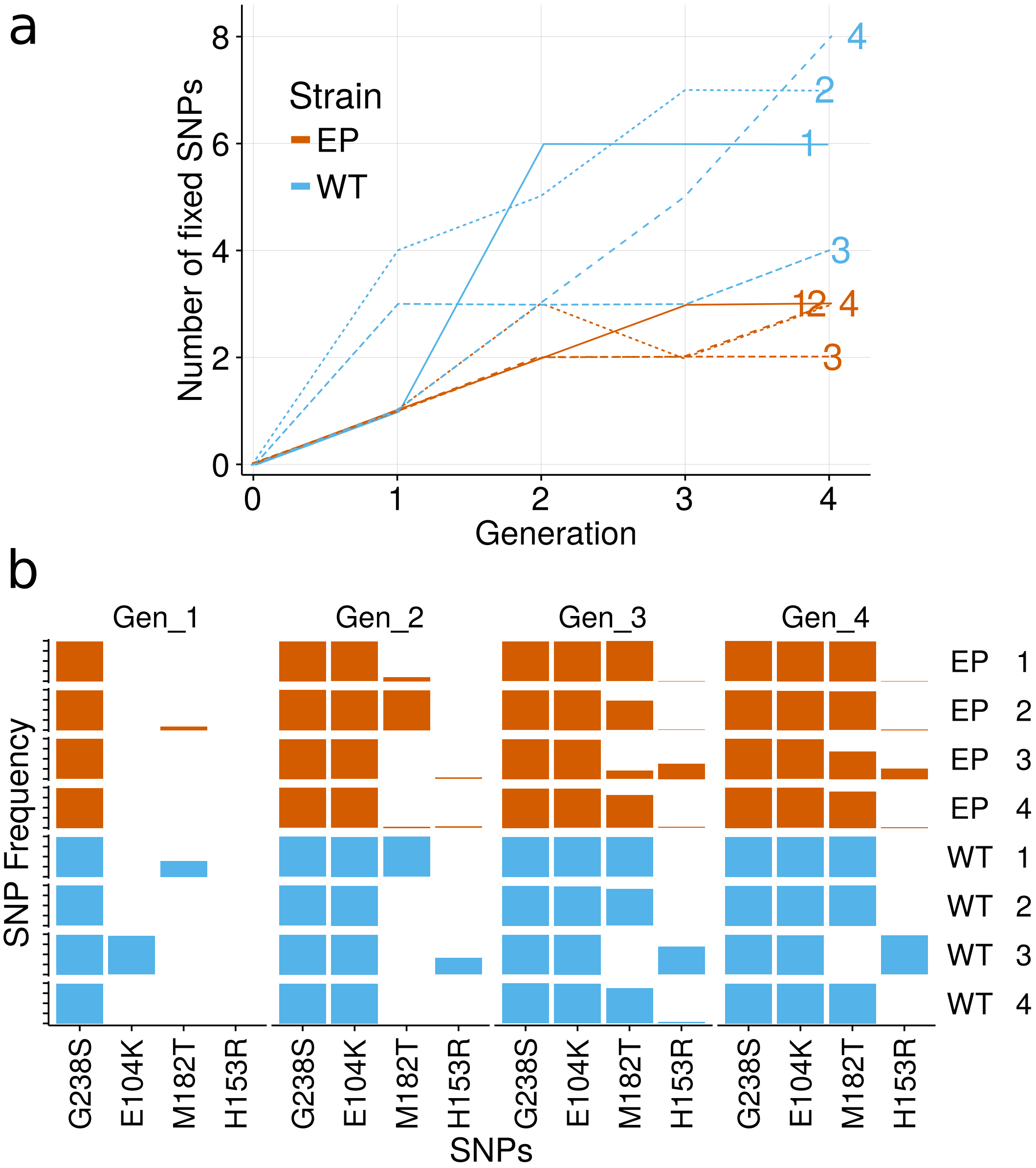

Supplementary Figure 3: Number of fixed SNPs, and frequencies of SNPs implicated in resistance to cefotaxime. **a**, The number of fixed SNPs (frequency greater than 90%) in experimental populations. The line type and the numbers correspond to the replicate population **b**, Frequency of SNPs known to be important for the evolution of cefotaxime resistance in all four generations of evolution. The height of the bar corresponds to the SNP frequency (shown in the range 0–100%).

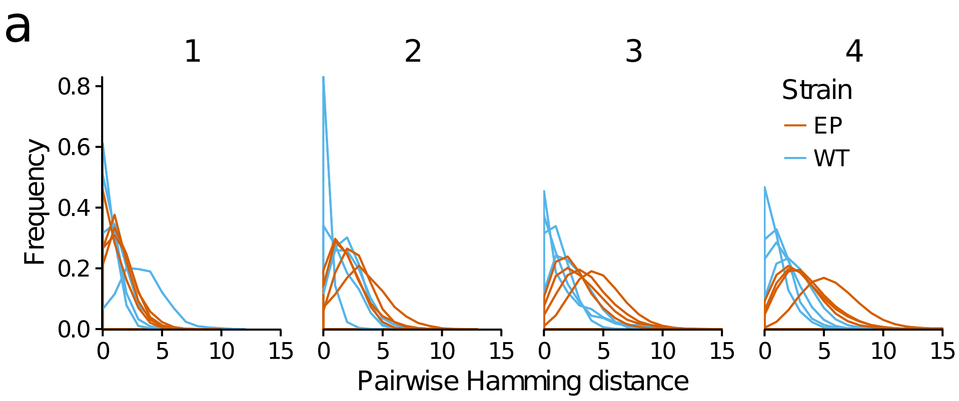

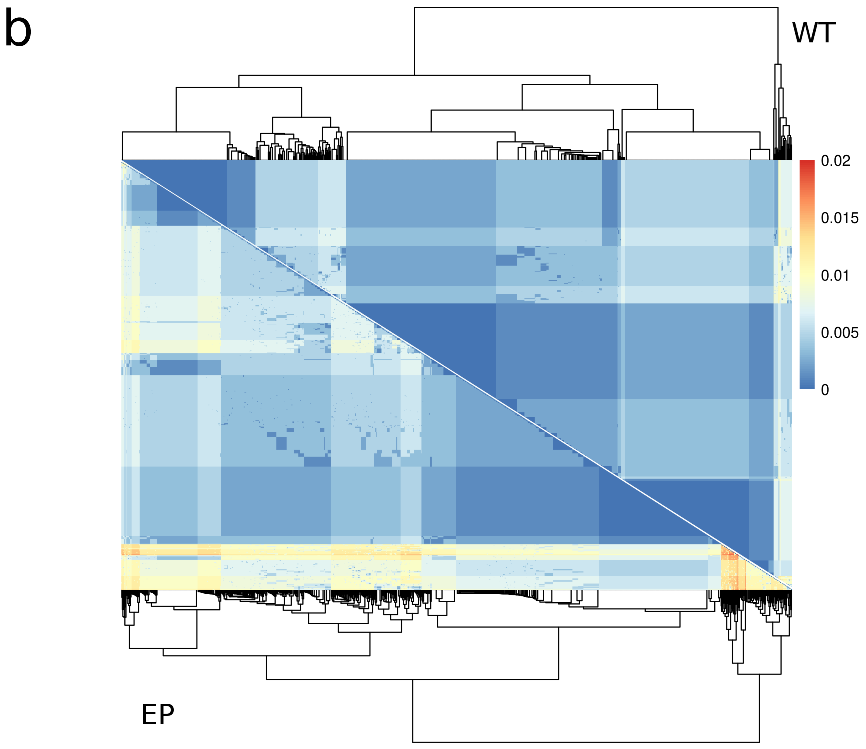

Supplementary Figure 4: Sequence diversity in evolved populations. **a**,The distribution of pairwise nucleotide sequence (Hamming) distances for each of the populations in all four generations. **b**, Diversity in pooled subsamples of nucleotide sequences from the final (fourth) generation. We randomly sampled 200 sequences from each of the populations, and then pooled them according to host in which they have evolved (EP = error-prone, WT = wild-type). We hierarchically clustered these sequences based on their nucleotide sequence identity, and created heatmaps of the resulting distance matrices. Pairwise sequence distance ranges from zero (blue) to 0.02 (red). The upper triangle corresponds to sequences from wild-type hosts, while the lower triangle corresponds to sequences from error-prone hosts.

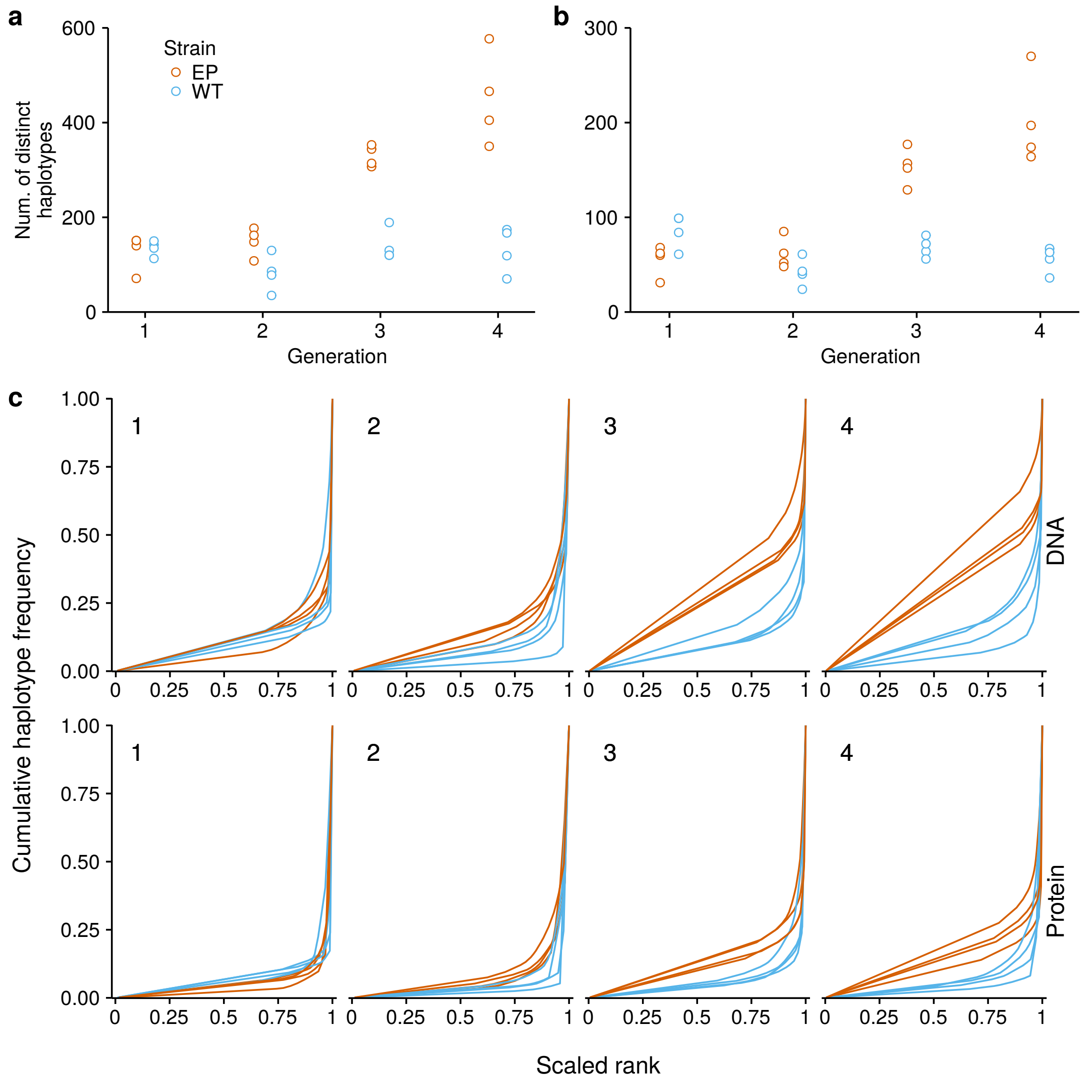

Supplementary Figure 5: Haplotype analysis in experimental populations. **a**, Number of distinct haplotypes (DNA level) in experimental populations. We counted the number of distinct haplotypes on the nucleotide level in each of the experimental populations during the four generations of experimental evolution. **b**, Number of distinct haplotypes (protein level) in experimental populations. We counted the number of distinct haplotypes on the protein level in each of the experimental populations during the four generations of experimental evolution. **c**, Cumulative variant (haplotype) frequencies in experimental populations. We calculated the frequency of each variant, on the DNA (top) and the protein (bottom) level, found in each of the populations from all four generations of evolution. We ranked variants based on their frequency, scaled their rank to an interval $[0, 1]$ range, and calculated the cumulative frequency distribution for each of the populations. For example, the cumulative frequency of 0.25 and the scaled rank of 0.9 mean that the frequencies of the 90% of all haplotypes (ordered by their abundance) sum up to 25%.

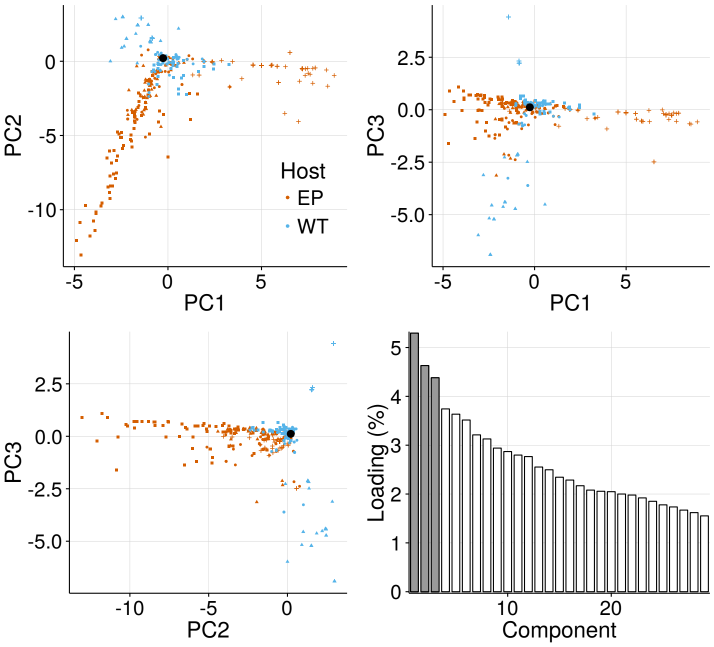

Supplementary Figure 6: The distribution of evolved DNA sequences in sequence space. We randomly sampled 200 sequences without replacement from all populations after the fourth generation of evolution, aligned them, and then projected the aligned sequences onto two dimensional space using principal component analysis (PCA). The figure shows the first three principal components (PC1, PC2, and PC3). Each symbol shape corresponds to a sequence, colors correspond to hosts (red = error-prone, blue = wild-type), and different shapes correspond to different replicate populations. The black circle corresponds to the ancestral TEM-1 sequence. The histogram shows axes loadings[9] for the first 30 principal component axes. The shaded region corresponds to the three axes used in the plot.

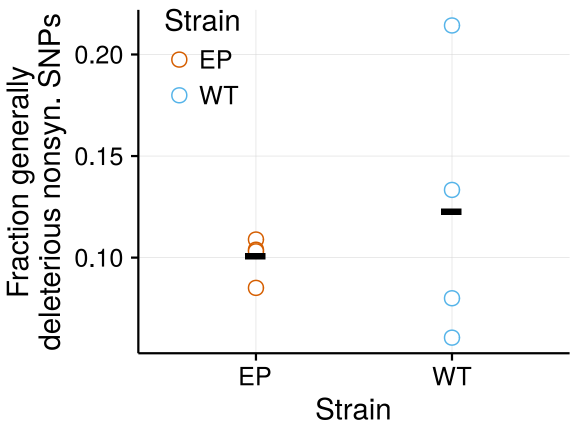

Supplementary Figure 7: Fraction of generally deleterious SNPs. Data are based on all nonsynonymous SNPs whose frequency decreases by more then 0.5% in both hosts and after selection in all three antibiotics (piperacillin, cefoxitin, and oxacillin with clavulanic acid).

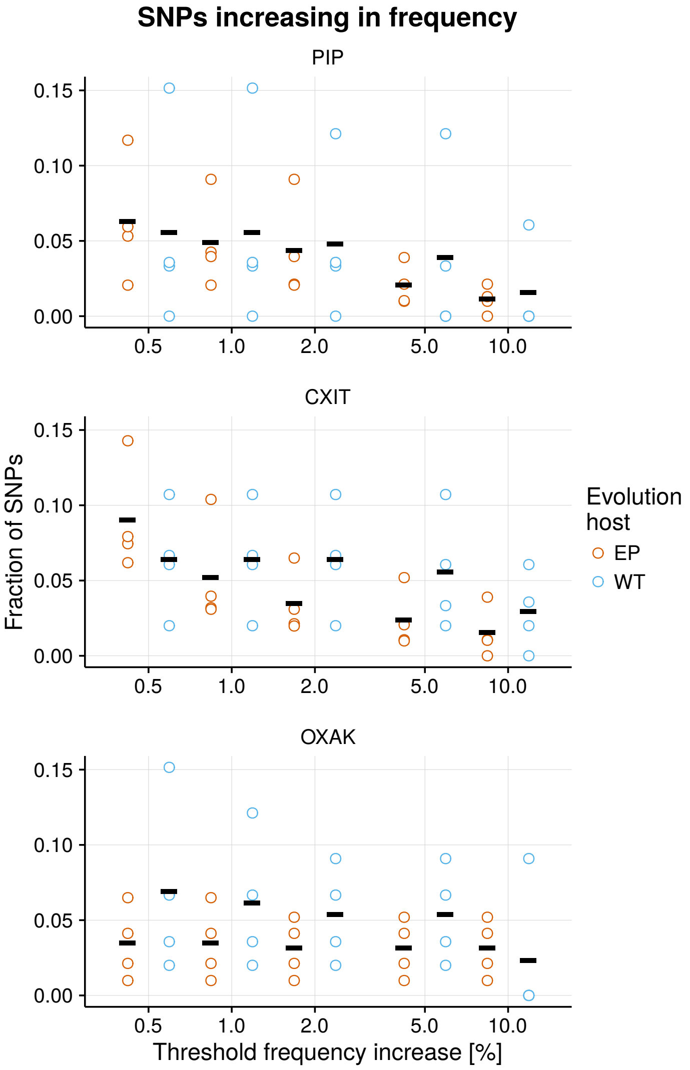

Supplementary Figure 8: Fraction of beneficial SNPs. The fraction of nonsynonymous SNPs whose frequency increases in wild-type hosts by more than 0.5% after selection in piperacillin (PIP), cefoxitin (CXIT), and oxacillin with clavulanic acid (OXAK).

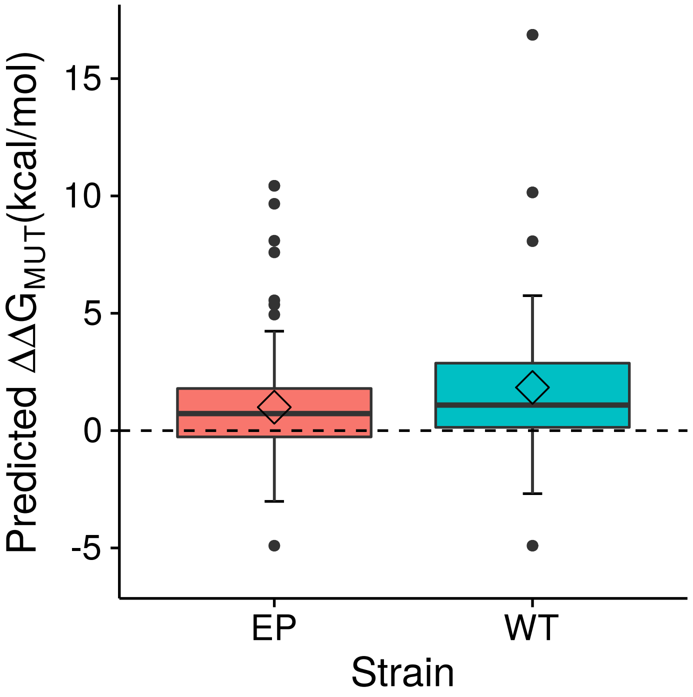

Supplementary Figure 9: Computationally predicted stability effects of purged SNPs. The figure shows the FoldX-predicted[10] distribution of $\Delta\Delta G_{MUT}$ for SNPs whose frequency decreased after the last generation of selection in cefotaxime. Mutations with lower $\Delta\Delta G_{MUT}$ values are less destabilizing. SNPs that decrease in frequency upon selection with cefotaxime between generation 3 and 4, have significantly lower $\Delta\Delta G_{MUT}$ in error-prone populations (two sided Mann–Whitney U test, U = 9507.5, P = 0.004649). Diamond shapes and thick horizontal lines show the mean and median of the distribution. Boxes extend to the first and third quartiles. The upper and the lower whiskers extend to the highest and the lowest values that are within 1.5 x IQR (inter-quartile range) of the box. Extreme values of $\Delta\Delta G_{MUT}$ (lower than -20 kcal/mol, and greater than 20 kcal/mol) were excluded from the plot for clarity, but they were included in the statistical analysis above.

Supplementary Tables

Supplementary Table 1: Cefotaxime MIC values after the selection. Values are in μg/mL. MIC(Ancestral) is the mean $\pm$ standard deviation of four replicates, measured for mistranslating and wild-type hosts carrying the pHS13T plasmid with the ancestral TEM-1, using the same medium as for experimental populations.

Strain	MIC(Ancestral)	Population	MIC(Gen1)	MIC(Gen2)	MIC(Gen3)	MIC(Gen4)
Mistranslating	0.055 ± 0.014	1	0.25	4.00	128.00	64.00
		2	0.25	4.00	64.00	32.00
		3	0.25	2.00	16.00	16.00
		4	0.25	4.00	32.00	16.00
Wild-type	0.141 ± 0.070	1	0.50	16.00	256.00	256.00
		2	1.00	8.00	128.00	256.00
		3	0.50	16.00	128.00	128.00
		4	0.50	4.00	64.00	256.00

Supplementary Table 2: Sequencing and SNP statistics. Population names (left-most column) are given in the format Host_Replicate_Generation. EP and WT refer to error-prone and wild-type hosts, respectively. The number of SNPs refers to observed SNPs before (raw) and after quality filtering (HQ).

Library	Reads	Mean Quality	SNPs (raw)	SNPs (HQ)	SNPs per read (raw)	SNPs per read (HQ)
EP_L1_G1	661	40.7	1409	1197	2.13	1.81
EP_L2_G1	723	40.7	1599	1258	2.21	1.74
EP_L3_G1	691	40.8	1182	1019	1.71	1.47
EP_L4_G1	734	40.9	1532	1230	2.09	1.68
EP_L1_G2	707	40.6	3321	2883	4.70	4.08
EP_L2_G2	721	40.8	3037	2782	4.21	3.86
EP_L3_G2	823	40.6	3676	3338	4.47	4.06
EP_L4_G2	671	40.6	2618	2275	3.90	3.39
EP_L1_G3	645	40.6	3089	2708	4.79	4.20
EP_L2_G3	673	40.5	3329	2891	4.95	4.30
EP_L3_G3	584	40.5	3361	2921	5.76	5.00
EP_L4_G3	705	40.4	4961	4408	7.04	6.25
EP_L1_G4	626	40.4	3290	2748	5.26	4.39
EP_L2_G4	698	40.6	3840	3225	5.50	4.62
EP_L3_G4	785	40.4	5422	4531	6.91	5.77
EP_L4_G4	896	40.4	6814	6130	7.60	6.84
WT_L1_G1	666	40.8	2755	2305	4.14	3.46
WT_L2_G1	743	40.9	3242	3104	4.36	4.18
WT_L3_G1	750	40.9	2550	2394	3.40	3.19
WT_L4_G1	755	40.8	2437	2218	3.23	2.94
WT_L1_G2	722	40.8	4379	4308	6.07	5.97
WT_L2_G2	766	40.7	4920	4605	6.42	6.01
WT_L3_G2	856	40.6	4007	3635	4.68	4.25
WT_L4_G2	751	40.7	3765	3549	5.01	4.73
WT_L1_G3	821	40.5	5526	5251	6.73	6.40
WT_L2_G3	786	40.7	7022	6732	8.93	8.56
WT_L3_G3	758	40.5	3861	3486	5.09	4.60
WT_L4_G3	767	40.6	6063	5835	7.90	7.61
WT_L1_G4	708	40.6	5017	4726	7.09	6.68
WT_L2_G4	750	40.6	7008	6693	9.34	8.92
WT_L3_G4	736	40.4	4301	3861	5.84	5.25
WT_L4_G4	737	40.7	7288	7090	9.89	9.62
EP_L1_CTRL	689	40.7	779	513	1.13	0.74
EP_L2_CTRL	767	40.6	905	590	1.18	0.77
WT_L1_CTRL	735	40.7	862	597	1.17	0.81
WT_L2_CTRL	740	40.4	900	617	1.22	0.83
TEM-1(Ancestor)	767	40.8	49	27	0.06	0.04
TEM-1(Ancestor)	800	40.6	50	31	0.06	0.04

Supplementary Table 3: Nonsynonymous SNPs found at frequencies greater than 10%. Rows are ordered according to position in TEM-1 (in Ambler numbering[11]). SNPs found at frequencies above 90% in at least one population are shown in bold. SNPs known to have stabilizing effects[1,8] are highlighted in cyan.

				Frequency in generation			
Position	SNP	Strain	Population	1	2	3	4
13	**I13T**	WT	3	95.7	98.7	97.8	98.8
15	F15L	WT	2	0.0	0.0	77.1	85.7
16	F16L	WT	4	0.0	74.3	1.0	0.0
21	L21P	WT	4	77.4	75.9	3.1	0.0
34	**K34R**	WT	2	0.0	0.0	90.6	97.7
38	D38N	WT	3	0.0	19.3	16.9	19.3
47	**I47V**	EP	4	17.9	59.8	80.3	87.8
		WT	4	0.0	0.0	92.2	99.1
56	**I56V**	WT	2	98.3	98.2	98.7	97.5
100	**N100D**	WT	4	84.0	98.5	99.1	97.8
104	**E104K**	EP	1	0.0	98.2	98.1	97.4
			2	0.0	97.8	98.5	97.4
			3	0.0	98.2	97.6	98.0
			4	0.0	99.1	96.9	98.9
		WT	1	0.0	98.5	97.8	98.3
			2	0.0	98.3	97.3	98.8
			3	95.6	98.6	98.8	97.6
			4	0.0	98.7	98.0	98.4
112	H112Y	EP	1	0.0	10.2	0.0	0.0
			2	0.0	0.0	48.9	32.5
120	R120G	EP	4	10.0	12.7	4.4	4.6
140	T140A	EP	1	16.8	37.5	14.7	13.1
			2	12.2	10.7	10.8	6.9
141	T141A	EP	3	3.2	48.0	18.2	28.9
146	K146E	EP	1	0.5	10.9	0.0	0.3
147	E147G	EP	1	0.0	32.0	0.2	0.0
		WT	3	0.0	18.0	14.9	2.7
153	**H153R**	EP	3	0.0	3.4	37.8	25.9
		WT	3	0.0	41.0	68.7	96.9
	H153D	WT	1	20.3	0.0	0.0	0.0
154	N154S	WT	2	0.0	17.1	0.3	0.0
173	I173T	EP	2	0.0	12.7	0.1	0.1
182	**M182T**	EP	1	0.0	9.9	98.9	98.2
			2	8.8	98.7	72.7	95.7
			3	0.0	0.1	20.5	68.0
			4	0.0	3.0	80.9	90.3
		WT	1	38.8	98.9	97.8	99.6
			2	0.0	0.0	90.8	99.5
			4	0.0	0.0	87.1	98.8
208	**I208M**	WT	2	98.6	98.2	98.1	97.5
224	A224V	WT	3	0.0	0.0	13.1	26.1
238	**G238S**	EP	1	98.5	98.3	97.7	99.0
			2	98.6	99.4	97.9	97.7
			3	99.1	98.5	97.6	98.5
			4	98.1	99.3	97.6	98.5
		WT	1	98.6	99.3	98.4	98.9
			2	99.3	99.2	98.3	98.3
			3	98.6	98.5	98.0	98.1
			4	98.8	98.7	99.3	98.8
265	**T265M**	WT	1	20.9	99.6	99.0	98.9
268	S268G	EP	3	2.8	65.5	61.6	50.8
273	D273G	WT	4	0.0	0.0	0.0	82.9
288	**K288E**	WT	2	98.5	97.9	97.8	96.7

Supplementary Table 4: Synonymous SNPs found at frequencies greater than 10%. Rows are ordered according to position in TEM-1 (in Ambler numbering[11]). SNPs found at frequencies above 90% in at least one population are shown in bold.

				Frequency in generation			
Position	SNP	Strain	Population	1	2	3	4
15	TTT15TTC	WT	1	47.6	0.0	0.0	0.0
21	**CTT21CTG**	EP	3	8.4	4.1	25.0	21.1
		WT	4	1.6	11.2	90.9	95.0
24	TTT24TTC	WT	1	46.1	0.0	0.1	0.0
76	**CTA76CTG**	WT	4	0.0	0.0	85.5	97.7
83	CGT83CGC	WT	2	0.0	22.6	4.2	0.0
84	GTT84GTC	EP	4	0.0	4.6	67.5	71.7
		WT	3	0.0	0.0	1.6	17.4
91	**CTC91CTT**	WT	1	0.2	99.4	97.8	97.7
97	TAT97TAC	EP	3	3.8	35.9	16.6	23.2
98	TCT98TCC	EP	3	6.5	3.8	24.1	18.7
107	CCA107CCG	WT	4	0.0	0.0	0.0	88.6
115	**GAT115GAC**	WT	4	0.0	4.5	86.3	98.8
120	AGA120AGG	EP	3	0.0	14.8	6.2	13.1
122	TTA122TTG	EP	2	11.5	5.8	0.4	0.3
	TTA122CTA	WT	2	0.1	24.9	4.1	0.0
144	GGA144GGG	WT	2	0.0	6.0	84.0	89.1
157	GAT157GAC	EP	2	0.0	13.0	0.1	0.6
162	**CTT162CTC**	WT	1	42.5	97.5	96.8	96.6
170	AAT170AAC	WT	2	0.0	19.6	0.0	0.0
184	GCA184GCG	EP	3	0.0	12.6	10.4	6.2
199	CTT199CTC	EP	1	0.0	36.8	0.2	0.2
207	TTA207CTA	EP	1	0.0	10.5	0.3	0.5
219	CCA219CCG	WT	2	0.0	15.1	0.0	0.0
225	CTT225CTC	EP	3	4.1	2.1	18.0	11.7
235	TCT235TCC	EP	4	1.2	2.2	63.4	73.7
274	GAA274GAG	EP	4	1.7	2.2	64.4	70.8
279	ATC279ATT	WT	3	0.0	20.8	13.7	2.6

Supplementary Table 5: Sequencing and SNP statistics of populations selected on cefoxitin. EvoHost denotes the host in which evolution took place, and PhenHost denotes the 'retransformation' host, in which the phenotype was assessed. EP and WT refer to error-prone, and wild-type strains, respectively. MIC is the minimal inhibitory concentration in μg/mL.

Population	EvoHost	PhenHost	Antibiotic	MIC	Reads	SNPs Per Read
EP_1	EP	EP	CXIT	4	1937	4.65
EP_1	EP	WT	CXIT	4	1990	5.14
EP_2	EP	EP	CXIT	4	2016	4.72
EP_2	EP	WT	CXIT	2	1863	5.02
EP_3	EP	EP	CXIT	4	2086	5.37
EP_3	EP	WT	CXIT	4	2209	6.76
EP_4	EP	EP	CXIT	4	2129	6.92
EP_4	EP	WT	CXIT	8	1874	7.13
WT_1	WT	EP	CXIT	2	2078	7.06
WT_1	WT	WT	CXIT	4	1806	7.52
WT_2	WT	EP	CXIT	2	2084	8.79
WT_2	WT	WT	CXIT	8	1854	10.43
WT_3	WT	EP	CXIT	4	2155	5.03
WT_3	WT	WT	CXIT	4	1813	7.13
WT_4	WT	EP	CXIT	8	1825	8.08
WT_4	WT	WT	CXIT	8	1665	7.42

Supplementary Table 6: Sequencing and SNP statistics of populations selected on oxacillin + clavulunic acid. EvoHost denotes the host in which evolution took place, and PhenHost denotes the 'retransformation' host, in which the phenotype was assessed. EP and WT refer to error-prone, and wild-type strains, respectively. MIC is the minimal inhibitory concentration in μg/mL.

Population	EvoHost	PhenHost	Antibiotic	MIC	Reads	SNPs Per Read
EP_1	EP	EP	OXAK	512	1769	3.27
EP_1	EP	WT	OXAK	1024	1968	5.73
EP_2	EP	EP	OXAK	512	1896	3.56
EP_2	EP	WT	OXAK	2048	1958	3.81
EP_3	EP	EP	OXAK	512	1650	4.09
EP_3	EP	WT	OXAK	1024	2621	5.57
EP_4	EP	EP	OXAK	512	1589	5.83
EP_4	EP	WT	OXAK	2048	1716	11.59
WT_1	WT	EP	OXAK	512	1613	5.69
WT_1	WT	WT	OXAK	1024	2208	7.33
WT_2	WT	EP	OXAK	512	2013	7.88
WT_2	WT	WT	OXAK	1024	2093	9.39
WT_3	WT	EP	OXAK	256	1812	5.15
WT_3	WT	WT	OXAK	1024	2265	9.37
WT_4	WT	EP	OXAK	512	2109	7.20
WT_4	WT	WT	OXAK	1024	1843	7.82

Supplementary Table 7: Sequencing and SNP statistics of populations selected on piperacillin. EvoHost denotes the host in which evolution took place, and PhenHost denotes the 'retransformation' host, in which the phenotype was assessed. EP and WT refer to error-prone, and wild-type strains, respectively. MIC is the minimal inhibitory concentration in μg/mL.

Population	EvoHost	PhenHost	Antibiotic	MIC	Reads	SNPs Per Read
EP_1	EP	EP	PIP	512	2095	4.16
EP_1	EP	WT	PIP	1024	1674	4.68
EP_2	EP	EP	PIP	512	2287	4.31
EP_2	EP	WT	PIP	1024	1913	4.37
EP_3	EP	EP	PIP	512	2145	5.33
EP_3	EP	WT	PIP	1024	2028	5.49
EP_4	EP	EP	PIP	512	2236	6.76
EP_4	EP	WT	PIP	1024	1932	6.65
WT_1	WT	EP	PIP	512	1971	6.38
WT_1	WT	WT	PIP	1024	2085	6.52
WT_2	WT	EP	PIP	512	1836	8.50
WT_2	WT	WT	PIP	1024	1915	8.71
WT_3	WT	EP	PIP	256	1931	5.00
WT_3	WT	WT	PIP	512	1659	5.19
WT_4	WT	EP	PIP	512	1827	8.00
WT_4	WT	WT	PIP	1024	1933	8.11

Supplementary Table 8: Primers and barcodes used for mutagenesis and sequencing

Primer	Sequence	Barcode
BC01	GGTAGGAGCAATGTAAAACGACGGCCAGT	AGCAAT
BC02	GGTAGGCCTGTTGTAAAACGACGGCCAGT	CCTGTT
BC03	GGTAGGGGGTTTGTAAAACGACGGCCAGT	GGGTTT
BC04	GGTAGGGAAGGCGTAAAACGACGGCCAGT	GAAGGC
BC09	GGTAGGTTAGGTGTAAAACGACGGCCAGT	TTAGGT
BC10	GGTAGGGTGCATGTAAAACGACGGCCAGT	GTGCAT
BC11	GGTAGGAACTTTGTAAAACGACGGCCAGT	AACTTT
BC12	GGTAGGGGATCGGTAAAACGACGGCCAGT	GGATCG
BC13	GGTAGGATAAGGGTAAAACGACGGCCAGT	ATAAGG
BC14	GGTAGGATTGGTGTAAAACGACGGCCAGT	ATTGGT
BC15	GGTAGGAGTGAGGTAAAACGACGGCCAGT	AGTGAG
BC16	GGTAGGCCCACCGTAAAACGACGGCCAGT	CCCACC
BC21	GGTAGGAACCTGGTAAAACGACGGCCAGT	AACCTG
BC22	GGTAGGCTTTGCGTAAAACGACGGCCAGT	CTTTGC
BC23	GGTAGGTGGAGAGTAAAACGACGGCCAGT	TGGAGA
BC24	GGTAGGAATTGTGTAAAACGACGGCCAGT	AATTGT
BC25	GGTAGGTGACGAGTAAAACGACGGCCAGT	TGACGA
BC27	GGTAGGGTTCAGGTAAAACGACGGCCAGT	GTTCAG
BC28	GGTAGGCTTCAAGTAAAACGACGGCCAGT	CTTCAA
TEM1FS-F	GTAAAACGACGGCCAGTGAATAATATTGAAAAAGGAAGC	-
TEM1FS-R	CAAGCAGAAGACGGCATACGAGCTCTTCCGATCTGTAAACTTGGTCTGACAGGAGC	-
ELP	GGTAGGCAAGCAGAAGACGGCAT	-
TEM-F6	GCTTAAGAATAATATTGAAAAAGG	-
TEM-R6	GAATTGTAAACTTGGTCTGACA	-

Supplementary Table 9: Concentrations of beta-lactam antibiotics used to test antibiotic susceptibility of evolved TEM-1 populations. All concentrations are given in in μg/mL. The concentration of clavulanic acid was 0.1 μg/mL in combination with cefotaxime, and 0.5 μg/mL in combination with oxacillin.

Cefotaxime	Cefotaxime + clavulanic acid	Ceftazidime	Cefoxitin	Oxacillin + clavulanic acid	Piperacillin
0.0625	0.0156	0.25	0.0156	2	2
0.125	0.0312	0.5	0.0312	4	4
0.25	0.0625	1	0.0625	8	8
0.5	0.125	2	0.125	16	16
1	0.25	4	0.25	32	32
2	0.5	8	0.5	64	64
4	1	16	1	128	128
8	2	32	2	256	256
16	4	64	4	512	512
32	8	128	8	1024	1024
64	16	256	16	2048	2048

Supplementary Table 10: Median minimal inhibitory concentrations (MIC) of six beta-lactam antibiotics for TEM-1 populations expressed in error-prone hosts. EvoHost denotes the host in which evolution took place. All concentrations are given in in μg/mL. CTX = Cefotaxime, CTXK = Cefotaxime + 0.1 μg/mL clavulanic acid, CTZ = Ceftazidime, CXIT = Cefoxitin, OXAK = Oxacillin + 0.5 μg/mL clavulanic acid, PIP = Piperacillin.

Population	EvoHost	CTX	CTXK	CTZ	CXIT	OXAK	PIP
Ancestral TEM-1	-	0.0625	0.0312	0.5	4	2048	512
EP_1	EP	16	0.125	64	4	256	256
EP_2	EP	16	0.125	64	4	256	256
EP_3	EP	16	0.125	64	8	256	256
EP_4	EP	16	0.125	64	6	256	256
WT_1	WT	16	0.125	32.0	8	256	256
WT_2	WT	16	0.125	64	8	256	256
WT_3	WT	8	0.0625	16	8	256	128
WT_4	WT	16	0.125	32	4	256	256

Supplementary Table 11: Median minimal inhibitory concentrations (MIC) of six beta-lactam antibiotics for TEM-1 populations expressed in wild-type hosts. EvoHost denotes the host in which evolution took place. All concentrations are given in in μg/mL. CTX = Cefotaxime, CTXK = Cefotaxime + 0.1 μg/mL clavulanic acid, CTZ = Ceftazidime, CXIT = Cefoxitin, OXAK = Oxacillin + 0.5 μg/mL clavulanic acid, PIP = Piperacillin.

Population	EvoHost	CTX	CTXK	CTZ	CXIT	OXAK	PIP
Ancestral TEM-1		0.0625	0.0625	0.5	8	> 2048	1024
EP_1	EP	16	0.2500	64	8	384	512
EP_2	EP	32	0.2500	64	8	512	512
EP_3	EP	16	0.2500	96	8	256	512
EP_4	EP	32	0.2500	64	8	384	512
WT_1	WT	32	0.2500	64	8	384	512
WT_2	WT	32	0.2500	128	8	512	512
WT_3	WT	8	0.1250	32	4	256	256
WT_4	WT	32	0.2500	64	8	256	512

Supplementary References

[1] Bratulic, S., Gerber, F. & Wagner, A. Mistranslation drives the evolution of robustness in TEM-1 β-lactamase. *Proceedings of the National Academy of Sciences* **112**, 201510071 (2015).

[2] Palzkill, T. & Botstein, D. Identification of amino acid substitutions that alter the substrate specificity of TEM-1 β-lactamase. *Journal of bacteriology* **174**, 5237–43 (1992).

[3] Orencia, M. C., Yoon, J. S., Ness, J. E., Stemmer, W. P. C. & Stevens, R. C. Predicting the emergence of antibiotic resistance by directed evolution and structural analysis. *Nature Structural Biology* **8**, 238–242 (2001).

[4] Weinreich, D. M., Delaney, N. F., Depristo, M. A. & Hartl, D. L. Darwinian evolution can follow only very few mutational paths to fitter proteins. *Science* **312**, 111 –114 (2006).

[5] Salverda, M. L. M. *et al.* Initial mutations direct alternative pathways of protein evolution. *PLoS Genetics* **7**, e1001321 (2011).

[6] Dellus-gur, E., Toth-Petroczy, A., Elias, M. & Tawfik, D. S. What makes a protein fold amenable to functional innovation? Fold polarity and stability tradeoffs. *Journal of Molecular Biology* **425**, 2609–21 (2013).

[7] Kather, I., Jakob, R. P., Dobbek, H. & Schmid, F. X. Increased folding stability of TEM-1 β-lactamase by *in vitro* selection. *Journal of Molecular Biology* **383**, 238–51 (2008).

[8] Salverda, M. L. M., de Visser, J. A. G. & Barlow, M. Natural evolution of TEM-1 β-lactamase: experimental reconstruction and clinical relevance. *FEMS Microbiology Reviews* **34**, 1015–36 (2010).

[9] Jolliffe, I. T. *Principal Component Analysis*. Springer Series in Statistics (Springer, 2002).

[10] Schymkowitz, J. *et al.* The FoldX web server: an online force field. *Nucleic Acids Research* **33**, W382–W388 (2005).

[11] Ambler, R. & Coulson, A. A standard numbering scheme for the class A β-lactamases. *Biochemical Journal* **276**, 269–272 (1991).
